# Purifying selection and low recombination facilitated sequential colonization of benthic and pelagic coastal ocean by ammonia-oxidizing archaea

**DOI:** 10.1093/ismeco/ycaf234

**Published:** 2025-12-08

**Authors:** Gaoyang Ren, Cécile Gubry-Rangin, Wenhao Wang, Ronghua Liu, Jiao Liu, Jinmei Liu, Xiao-Hua Zhang, Jiwen Liu

**Affiliations:** Frontiers Science Center for Deep Ocean Multispheres and Earth System, and College of Marine Life Sciences, Ocean University of China, Qingdao 266003, China; Laboratory for Marine Ecology and Environmental Science, Qingdao Marine Science and Technology Center, Qingdao 266237, China; Key Laboratory of Evolution & Marine Biodiversity (Ministry of Education) and Institute of Evolution & Marine Biodiversity, Ocean University of China, Qingdao 266003, China; School of Biological Sciences, University of Aberdeen, Aberdeen AB24 3UU, United Kingdom; Frontiers Science Center for Deep Ocean Multispheres and Earth System, and College of Marine Life Sciences, Ocean University of China, Qingdao 266003, China; Laboratory for Marine Ecology and Environmental Science, Qingdao Marine Science and Technology Center, Qingdao 266237, China; Key Laboratory of Evolution & Marine Biodiversity (Ministry of Education) and Institute of Evolution & Marine Biodiversity, Ocean University of China, Qingdao 266003, China; Frontiers Science Center for Deep Ocean Multispheres and Earth System, and College of Marine Life Sciences, Ocean University of China, Qingdao 266003, China; Laboratory for Marine Ecology and Environmental Science, Qingdao Marine Science and Technology Center, Qingdao 266237, China; Key Laboratory of Evolution & Marine Biodiversity (Ministry of Education) and Institute of Evolution & Marine Biodiversity, Ocean University of China, Qingdao 266003, China; Frontiers Science Center for Deep Ocean Multispheres and Earth System, and College of Marine Life Sciences, Ocean University of China, Qingdao 266003, China; Laboratory for Marine Ecology and Environmental Science, Qingdao Marine Science and Technology Center, Qingdao 266237, China; Key Laboratory of Evolution & Marine Biodiversity (Ministry of Education) and Institute of Evolution & Marine Biodiversity, Ocean University of China, Qingdao 266003, China; Frontiers Science Center for Deep Ocean Multispheres and Earth System, and College of Marine Life Sciences, Ocean University of China, Qingdao 266003, China; Laboratory for Marine Ecology and Environmental Science, Qingdao Marine Science and Technology Center, Qingdao 266237, China; Key Laboratory of Evolution & Marine Biodiversity (Ministry of Education) and Institute of Evolution & Marine Biodiversity, Ocean University of China, Qingdao 266003, China; Frontiers Science Center for Deep Ocean Multispheres and Earth System, and College of Marine Life Sciences, Ocean University of China, Qingdao 266003, China; Laboratory for Marine Ecology and Environmental Science, Qingdao Marine Science and Technology Center, Qingdao 266237, China; Key Laboratory of Evolution & Marine Biodiversity (Ministry of Education) and Institute of Evolution & Marine Biodiversity, Ocean University of China, Qingdao 266003, China; Frontiers Science Center for Deep Ocean Multispheres and Earth System, and College of Marine Life Sciences, Ocean University of China, Qingdao 266003, China; Laboratory for Marine Ecology and Environmental Science, Qingdao Marine Science and Technology Center, Qingdao 266237, China; Key Laboratory of Evolution & Marine Biodiversity (Ministry of Education) and Institute of Evolution & Marine Biodiversity, Ocean University of China, Qingdao 266003, China

**Keywords:** *Nitrososphaeria*, ammonia-oxidizing archaea, coastal ocean, niche transition, evolutionary adaptation

## Abstract

The evolutionary adaptation of archaea to ecologically diverse habitats remains poorly understood. Ammonia-oxidizing archaea (AOA) exhibit significant diversification across various environmental conditions; however, their ecological dynamics, diversification, and associated evolutionary processes are still largely unexplored in coastal environments, which contain extensive ecosystem heterogeneity. Combining newly assembled metagenomic data from Chinese marginal seas (2059 km coverage) with global datasets (spanning over 16 000 km), these knowledge gaps were explored across a continental-scale latitudinal gradient. It revealed that coastal AOA genomic diversity is latitude-dependent, with predicted optimum growth temperatures and substrate metabolic pathways explaining the geographical distribution. The two dominant genus-level clades exhibited significantly distinct benthic-pelagic niches, associated with specific genes involved in nutrient uptake and stress resistance. Phylogenomic reconstructions suggest that AOA initially colonized the coastal ocean sediments around 718 million years ago (Mya), and subsequent purifying selection and low recombination facilitated the AOA niche expansion into marine coastal environments. By revealing the evolutionary trajectories of *Nitrososphaeria* and their differential colonization patterns, our findings offer a novel perspective on the mechanisms of AOA diversification in the coastal ocean. This work advances our understanding of microbial diversification and niche differentiation of AOA in coastal ecosystems as well as the evolutionary forces shaping their global biogeography.

## Introduction

Ammonia-oxidizing archaea (AOA) play crucial roles in the global carbon and nitrogen cycles, serving as the primary ammonia oxidizers in the ocean [1, 2]. All AOA are assigned to the *Nitrososphaeria* class, previously known as *Thaumarchaeota* [3]. AOA inhabit diverse oxic environments within the marine ecosystem, from coastal to open ocean areas, and from surface water to deep sediments [3–5], thereby occupying a broad range of environmental niches. Diverse phylogenetic lineages sustain the widespread distribution of *Nitrososphaeria*, each exhibiting distinct spatial patterns and adaptive strategies [6]. Specifically, while *Nitrososphaeraceae* and *Nitrosocaldaceae* are prevalent in terrestrial habitats, *Nitrosopumilaceae* (NP) is the dominant group in the ocean. Moreover, niche partitioning has been observed among members of NP, with *Nitrosopumilus* being predominantly distributed in coastal areas, *Candidatus* Nitrosopelagicus (i.e. the water column A group) being prevalent in open ocean surface waters, and the water column B group being more common in deep-sea water [7–10]. Additionally, previous studies have demonstrated significant phylogenetic divergence of AOA between deep-sea water and deep-sea sediments [11–13]. In contrast, coastal seas, including both water and sediments, are predominantly inhabited by *Nitrosopumilus* [7, 14]. The extent to which niche separation and biogeography occur within this vast coastal AOA community is poorly understood, particularly given its considerable genetic diversity [6] and the significant environmental heterogeneity of coastal seas on a global scale.

AOA are thought to have originated in terrestrial environments and later expanded into the ocean, coinciding with the timing of ocean oxygenation [15]. Freshwater-marine boundaries are typically characterized by sharp salinity gradients, which act as strong environmental filters, limiting microbial dispersal and resulting in phylogenetic divergence that is often detectable even at higher taxonomic levels (e.g. phylum or class) [16–18]. Transitions across this salinity gradient have been infrequent throughout the evolutionary history of aquatic microbes [17, 19]. However, AOA have successfully crossed this barrier [20], accompanied by substantial evolutionary divergence [21]. This divergence from terrestrial AOA involved the acquisition of strategies to survive under higher osmotic pressures and lower temperatures [22, 23], thereby fostering the evolution of marine-specific lineages. The coastal area, as a crucial interface between land and ocean, was likely the habitat enabling the transition from terrestrial to marine environments. However, the processes by which these microorganisms colonized, established and diversified their communities remain largely unknown. Understanding these processes is essential for bridging the gap in our knowledge of niche transition-associated evolution in *Nitrososphaeria* and for understanding how they adapt to a wide range of environmental conditions.

In this study, we employed metagenomic sequencing in conjunction with comprehensive genomic and evolutionary analyses to investigate the distribution patterns and evolutionary trajectories of ammonia-oxidizing *Nitrososphaeria* inhabiting the coastal ocean. We initially characterized the diversity of *Nitrososphaeria* in sediments from the Chinese marginal seas, where environmental heterogeneity is high and *Nitrososphaeria* are the most abundant archaeal members [24]. We then explored the niche differentiation among distinct phylogenetic lineages of coastal *Nitrososphaeria* on a global scale. Furthermore, we assessed the evolutionary history across the different lineages and explored the driving forces underlying their evolution. We aimed to provide a comprehensive understanding of the mechanisms by which *Nitrososphaeria* successfully established its diverse communities in the coastal ocean from both ecological and evolutionary perspectives.

## Material and methods

### Sample collection and DNA extraction

Surface sediment samples (0–2 cm) were collected from 18 stations along the Chinese coastal line, covering the four marginal seas (Supplementary Fig. S1). These included one site in the Bohai Sea (B3), four sites in the Yellow Sea (HS5, HS12, YS03, and YS07), eight sites in the East China Sea including the Changjiang Estuary (A6-3, A6-7, C2, F2, H2, ECS06, ECS12, and ECS15), and five sites in the South China Sea (SCS01, SCS03, SCS06, SCS09, and SCS12). Before metagenomic sequencing, the sediment samples were placed in cryovials and stored at −80°C. Environmental parameters (e.g. temperature, salinity, dissolved oxygen, and inorganic nutrients) were measured at most sites, and the available data are summarized in Supplementary Table S1.

### Metagenomic assembly, genome binning, and taxonomic annotation

DNA extractions from the sediment samples were performed according to previously described methods [25]. Details of the DNA extractions can be found in the Supplementary Information. Shotgun metagenomic sequencing was performed using the Illumina HiSeq X Ten platform (2 × 150 bp paired-end), with an average output of ~28.5 Gbp per sample (Supplementary Table S2). Metagenomic data from stations HS5 and HS12 were obtained from a previously published study [26]. All sequencing data were processed and quality-controlled using Trimmomatic (v0.39) [27]. Subsequent assembly, binning, and refining were performed using the metaWRAP pipeline (v1.3.2) [28]. Metagenomes were assembled using MEGAHIT (v1.1.3) [29] and binned with a combination of CONCOCT (v1.0.0) [30], MetaBAT (v2.12.1) [31], and MaxBin2 (v2.2.6) [32] using default parameters and k-mers. The resulting bins were refined using the bin refinement module in metaWRAP (v1.3.2) and under anvi’o (v7.1) [33] for improved data quality before downstream analysis. Metagenome-assembled genomes (MAGs) completeness and contamination were assessed using CheckM (v1.2.2) [34]. To enhance phylogenetic resolution and preserve potentially meaningful genomic variation shaped by microenvironmental differences, we retained all MAGs regardless of redundancy, as they were recovered from distinct coastal sampling sites characterized by varying environmental conditions (Supplementary Table S3). Taxonomic classifications were obtained using the Genome Taxonomy Database Toolkit (GTDB-Tk v2.4.0) [35] with the GTDB Release R220 [36]. To enhance the integrity of the medium-quality and high-quality MAGs, two strategies were employed: merging and assembling data from adjacent stations within the same sampling area to address low MAG completeness, and splitting and reassembling data to reduce the impact of high read diversity, thereby ensuring higher quality.

### Relative abundance of metagenome-assembled genomes

We compiled a comprehensive dataset consisting of 360 metagenomes sourced from various environments: coastal sediments (*n* = 119), coastal waters (*n* = 116), open ocean waters (*n* = 48), deep-sea waters (*n* = 58), and deep-sea sediments (*n* = 19) (Supplementary Table S4). This dataset integrates metagenomes generated from this study, as well as 342 publicly available metagenomes from the NCBI database and the *Tara Oceans* project. The relative abundance of MAGs and reference genomes was estimated by individually mapping metagenomic reads to the MAGs using CoverM (v0.6.1; https://github.com/wwood/CoverM). Paired reads with alignment identities over 99% and a minimum alignment length of 50 nt were collected, sorted, and used to calculate relative abundance with the “-m relative_abundance” parameter in CoverM (v0.6.1).

### Phylogenetic inferences and pangenome analysis

The AOA phylogeny was inferred from 13 reconstructed MAGs and 72 reference genomes from diverse habitats, focusing on coastal environments (Supplementary Table S5). Additionally, 16S rRNA gene sequences were extracted from coastal sediment metagenomes, clustered at 97% similarity, and aligned with reference sequences. Phylogenies of key functional genes (e.g. *amoA*, *ureC*, *pstB*, and the gene encoding chitinase) were inferred to explore metabolic diversity. Further details on methods, software, and parameters are provided in Supplementary information, under “Material and Methods: Phylogenetic inferences.”

MAGs and reference genomes with completeness >80% and contamination <5% were selected for core and pan-genome analysis. Specifically, gene coding sequences of genomes were predicted by Prokka (v1.14.6; default parameters) [37]. Orthologous gene families among all genomes were identified using Orthofinder (v2.5.4) [38]. The core genome comprises orthogroups shared by all MAGs, while the pangenome encompasses the total set of genes present in at least one genome. For *m* selected out of *n* genomes, a total of *n*!/[*m*!·(*n*–*m*)!] combinations were calculated to determine the sizes of the core and pangenomes. Pangenomic comparison was performed using anvi’o (v7.1) following the available tutorial (https://merenlab.org/2016/11/08/pangenomics-v2/) as well as guidance from a previous study [33].

### Genome and proteome characteristics

Average nucleotide identity (ANI) between MAGs was calculated using pyani (https://github.com/widdowquinn/pyani; v0.2.12), and average amino acid identity (AAI) across genomes was determined using CompareM (https://github.com/dparks1134/CompareM; v0.1.2). Genome size, GC content, and coding sequence number and density were assessed in CheckM (v1.2.2) [34]. Optimal growth temperature was predicted based on genomes using CnnPOGTP [39]. The numbers of carbon and nitrogen atoms per amino acid residue side chain (C-ARSC and N-ARSC) were calculated using a Perl script (https://github.com/ylifc/aoa-in-methane-seep-sediment). Differences in amino acid composition and codon usage were calculated by CompareM (https://github.com/dparks1134/CompareM; v0.1.2). Additional genome functional annotation and metabolic analysis are described in the Supplementary Information.

### Estimation of species divergence times

The divergence time of *Nitrososphaeria* was assessed using MCMCTree (burnin = 800 000, sample frequency = 10, number of samples = 200 000). Analysis was performed on the maximum-likelihood tree inferred from the concatenated 122 conserved archaeal marker proteins. Due to the absence of molecular fossil records for *Nitrososphaeria*, four constraints from other archaea were used [23]: the archaea root of 4380–3460 Mya [40], the *Crenarchaeota Sulfolobales* and *Thermoproteales* last common ancestor constraints at 2330 Mya [41], and the most recent common ancestor of *Crenarchaeota Sulfolobus solfataricus* and *Sulfolobus islandicus* at ~475 Mya [42]. The tree was rooted using members of the DPANN lineage, which has been proposed as a potential early-diverging group [43]. To assess robustness, we repeated the analysis by rooting the tree with members of the *Euryarchaeota* lineage. The two rooting schemes yielded comparable estimates of divergence time.

### Estimation of evolutionary metrics

Filtered reads from each compiled metagenomic dataset (*n* = 360) were mapped to the concatenated representative MAG of each species cluster, derived from the 13 reconstructed MAGs and 72 reference genomes, using Bowtie2 (v2.2.5) with default parameters [44]. Population statistics and nucleotide metrics, including linkage disequilibrium (*D*′), single-nucleotide variant density (SNVs/kbp), nonsynonymous to synonymous mutation ratio (*pN/pS*), and nucleotide diversity, were calculated at the genome level using the profile module of inStrain (v1.3.1; −database mode; default parameters) [45]. Genetic annotation of MAGs was performed with Prodigal (v2.6.3; –p meta) [46] for the gene module of inStrain. For SNP calling, the number of quality-filtered reads mapping to the position had to be at least 5 × coverage and 5% SNP frequency, with the variant reads exceeding the expected sequencing error rate (1 × 10^−6^).

## Results

### Coexistence of phylogenetically diverse *Nitrososphaeria* clusters in Chinese coastal sediments

Eighteen sediment metagenomes spanning a spatial distance of 2095 km along the Chinese marginal seas were analyzed. The *Nitrososphaeria* 16S rRNA gene sequences (excluding those of non-AOA) were extracted from the metagenomes and were clustered into 21 representative sequences at a similarity threshold of 97% to determine species diversity in these coastal sediments. These sequences were predominantly affiliated with *amoA*-NP-γ (97.4%), followed by *amoA*-NP-θ (0.85%) and *amoA*-NP-δ (0.85%), based on a reference framework that links 16S rRNA gene taxonomy to *amoA*-defined clades through co-occurring sequences in reference genomes, as proposed by Wang *et al.* [6, 47]. Finer classification revealed a high diversity within *amoA*-NP-γ (Supplementary Figs. S2 and S3), with NP-γ-2.2.2.1 (75.4%) being the dominant clade, which encompasses sequences previously recovered mainly from estuarine/coastal sediments and waters [48].

Metagenome binning generated 13 *Nitrososphaeria* MAGs from the coastal sediments, including five from the Bohai Sea and eight from sites close to the Changjiang estuary (Supplementary Table S5). The retrieved genomes had a median completeness of 80.9% (50.2%–92.2%) and a contamination level of 2.9% (0%–9.7%), with genome sizes ranging from 0.76 to 1.97 Mbp. They were affiliated with four genera of the NP family, including *Nitrosopumilus* (*n* = 6), JACEMX01 (*n* = 5), PXYB0l (*n* = 1), and CSP1-1 (*n* = 1). Similarly, phylogenomic analyses based on 54 ribosomal proteins placed the genomes into four highly supported clusters (Supplementary Fig. S4). The genomes belonging to *Nitrosopumilus* and JACEMX01 formed a well-supported monophyletic lineage, together with other reference AOA genomes from coastal environments. Members of JACEMX01 (e.g. S1bin1 and S2bin1) have been delineated as a novel species of *Nitrosopumilus* [48]. However, calculation of ANI and AAI values, including the newly retrieved genomes, showed that JACEMX01 represents a distinct genus, with pairwise AAI values of 72.8%–74.7% and ANI values of 84.5%–90.0% relative to *Nitrosopumilus* genomes (Supplementary Fig. S5), consistent with a recent study naming this genus *Candidatus* Nitrosomaritimum [49]. The 16S rRNA gene was annotated in only one of the MAGs (A6-3-1bin3) and was affiliated to NP-γ-2.1.3.1 (Supplementary Fig. S6). The remaining MAGs were classified based on the 16S rRNA gene classification of their closest phylogenetic neighbors (ANI > 95.8%), which showed affiliations of the *Nitrosopumilus*-related genomes to NP-γ-2.1.3 (*n* = 7), and JACEMX01-related genomes to NP-γ-2.2.2.1 (*n* = 7), respectively (Supplementary Fig. S6). This approach enabled us to relate the MAGs to established functional lineages of AOA [6, 50].

### Global biogeography of different coastal benthic clusters

**Figure 1:**
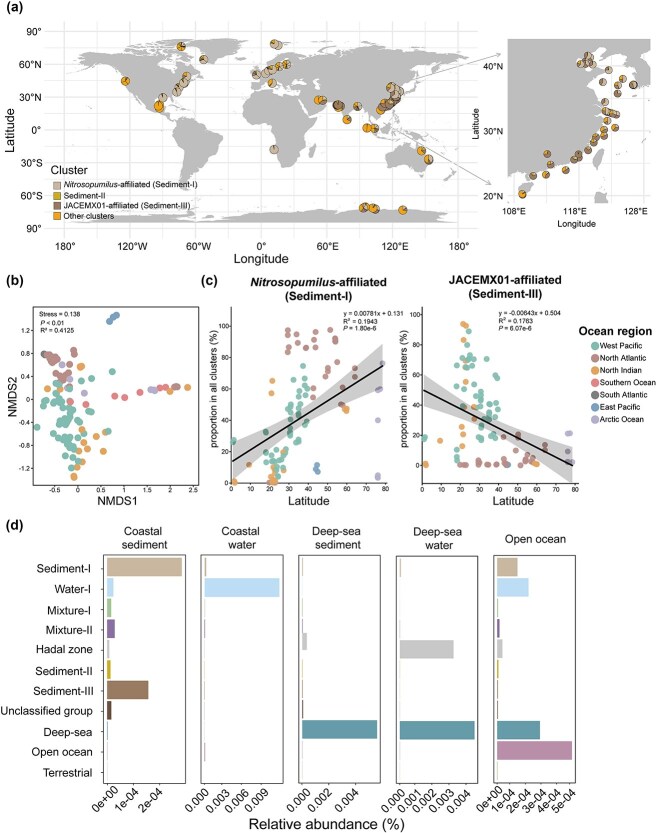
Ecological distribution of distinct *Nitrososphaeria* clusters across various environmental settings. (a) Reads mapping showing the relative distribution of the coastal sediment-derived MAGs across global coastal sediment samples. (b) Ordination of communities using the non-metric multidimensional scaling based on Bray–Curtis dissimilarities, with each point representing an individual sample of coastal sediment. (c) Correlation between the distribution of the two dominant sediment clusters with latitude in coastal sediments from the northern hemisphere, with a linear regression fitted and Pearson correlation applied. (d) Median relative abundance of distinct *Nitrososphaeria* clusters, calculated from metagenomic read recruitment to MAGs of each cluster shown in Fig. 2, across various ecological environments (see also Supplementary Fig. S11).

**Figure 2:**
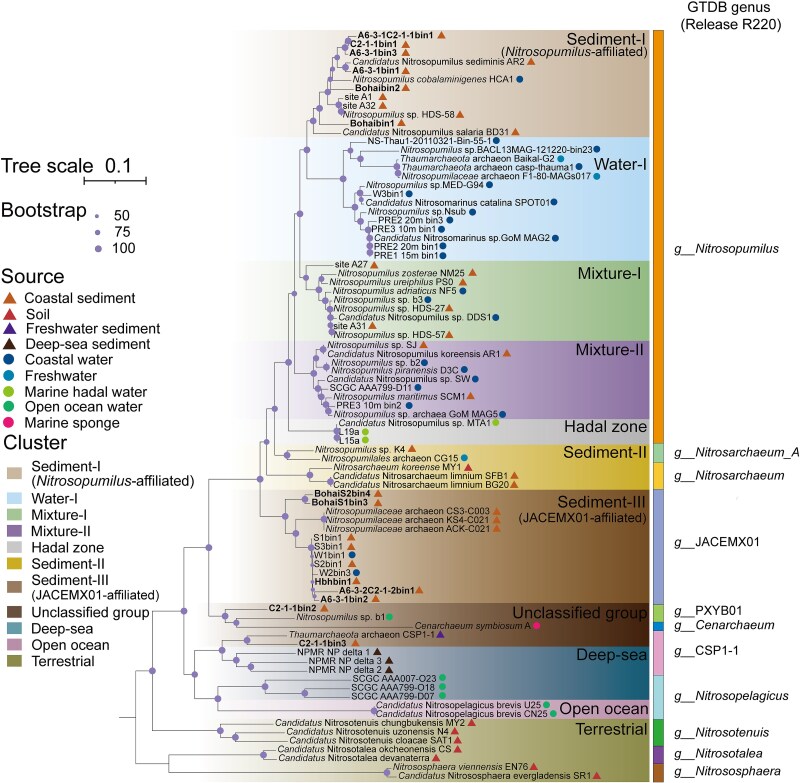
Maximum-likelihood phylogeny of *Nitrososphaeria* based on 54 ribosomal proteins, constructed using IQ-TREE (v2.2.2.6) with the LG+F+R5 model and 1000 bootstrap replicates. The environmental origins of MAGs generated in this study (highlighted in bold) and closely related reference genomes are indicated to the right of the respective branch tips.

Among the newly reconstructed genomes, competitive read recruitment showed a higher abundance of genomes belonging to *Nitrosopumilus* and JACEMX01 compared to those of other genera in sediments of the Chinese marginal seas (*P* < .01, Supplementary Fig. S7). These genera also dominated when multiple coastal sediments (*n* = 119) from a global scale were included (Fig. 1a). In 79.0% of the worldwide sediment samples, either *Nitrosopumilus*- or JACEMX01-affiliated genomes represented the most abundant groups, jointly accounting for an average of 78.8% ± 15.2% (range: 37.6%–99.0%) of the *Nitrososphaeria* population. In sediment samples dominated by various other *Nitrososphaeria*-affiliated groups not classified as *Nitrosopumilus*-affiliated or JACEMX01-affiliated cluster (see other groups in Fig. 2), 10.9% were dominated by groups from the deep sea, and 2.5% by those from coastal/shallow seawater. Thus, *Nitrosopumilus*- and JACEMX01-affiliated lineages dominated the *Nitrososphaeria* population in global coastal sediments.

**Figure 3:**
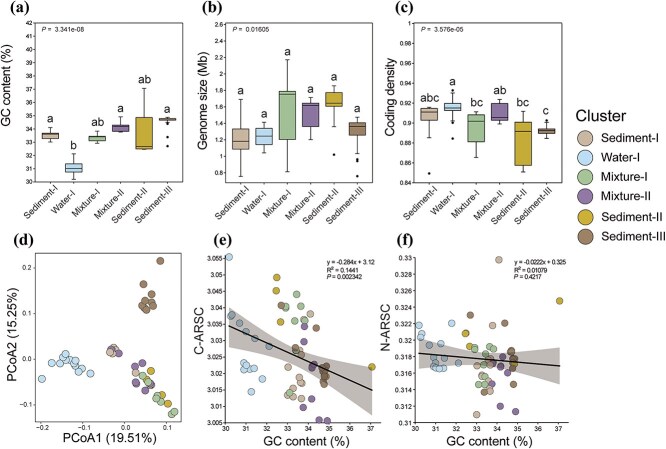
Comparison of genomic and proteomic characteristics among the six major, well-supported coastal *Nitrososphaeria* clusters, with assessments of (a) GC content, (b) genome size, and (c) coding density. (d) Similarity of predicted proteomic compositions based on orthologous genes across different clusters. (e and f) Relationship between average carbon (C-ARSC) and nitrogen (N-ARSC) atoms per amino acid residual chains with the GC content, with a linear regression fitted and Pearson correlation applied.

The two dominant clusters of the coastal benthic *Nitrososphaeria* are geographically distributed according to global sampling geographical zones (PERMANOVA: *R*^2^ = 0.413, *P* < .01; Fig. 1b). Both *Nitrosopumilus* and JACEMX01 displayed significant, yet opposing, latitude-dependent distribution, particularly in the northern hemisphere, where more datasets were collected (Fig. 1a). The *Nitrosopumilus*-affiliated cluster increased in relative abundance with latitude, peaking between ~30°N and 60°N in the coastal regions of the North Atlantic (Spearman’s rank correlation, *P* < .001). Conversely, the JACEMX01-affiliated cluster exhibited a decreasing trend with increasing latitude, with its highest abundance between 20°N to 30°N in the coastal regions of the Indian Ocean and Western Pacific (Spearman’s rank correlation, *P* < .001) (Fig. 1c). Correspondingly, the predicted optimal growth temperatures of the JACEMX01-affiliated cluster were higher than those of the *Nitrosopumilus*-affiliated cluster (Wilcoxon test, *P* < .05, Supplementary Fig. S8).

### Phylogeny-related niche partitioning between coastal water and sediments

Given the dominance of *Nitrosopumilus* in both coastal water and sediments, we further investigated the potential niche partitioning between these two habitats in *Nitrosopumilus* populations. Phylogenomic analysis with reference genomes revealed the division of *Nitrosopumilus* into six well-supported clusters, validated by their distinct ANI and AAI values (Supplementary Fig. S5). This clustering pattern was consistent across phylogenies based on 54 ribosomal proteins (using both maximum likelihood and Bayesian algorithms; Supplementary Table S6, Supplementary Figs. S4 and S9a) and 122 single-copy genes (using maximum-likelihood algorithms; Supplementary Fig. S9b). A strict consensus tree confirmed the robustness of this clustering pattern by consistently revealing the same branching structures across all datasets. Two *Nitrosopumilus* phylogenetic clusters exhibited notable niche preferences (Fig. 2), with one cluster, designated “Sediment-I,” containing genomes primarily originating from sediments (including those from this study), whereas the other, designated “Water-I,” consisted mainly of genomes from seawater. Additionally, two other *Nitrosopumilus* phylogenetic clusters (“Mixture-I” and “Mixture-II”) comprised genomes originating from seawater and sediment sources. Finally, *Nitrosarchaeum* and JACEMX01 each encompassed a sediment-dominated cluster named “Sediment-II” and “Sediment-III,” respectively (Fig. 2).

To further verify the phylogeny-related environmental preference, a competitive read recruitment approach was performed to compare the relative distribution of distinct clusters between coastal waters and sediments globally (Fig. 1d, Supplementary Figs. S10 and S11). The Sediment-I and Sediment-III clusters were highly abundant in coastal sediments compared to other habitats (Wilcoxon test, *P* < .01; Fig. 1d), whereas the Water-I cluster predominated in the coastal water (Wilcoxon test, *P* < .05; Fig. 1d). The presence of Sediment-I and Water-I clusters in the open ocean water likely reflected their niche expansion. The open-ocean and deep-sea clusters were most abundant in the environments from which they originated. These findings confirmed that water-*versus*-sediment niche partitioning led to phylogenetic diversification in coastal-derived *Nitrosopumilus*.

### Genomic features and metabolic adaptation associated with niche separation

Genomes affiliating to the Water-I cluster had the smallest genome size (1.04–1.41 Mbp) and the lowest GC content (30%–32%) among the tested clusters (Fig. 3a and b). These traits, coupled with higher coding density compared to sediment-preferred clusters (Kruskal-Wallis test, *P* < .05; Fig. 3c), suggest a process of genome streamlining. In contrast, genomes affiliating to the Sediment-III cluster had the highest GC content (33%–35%, Fig. 3a). Furthermore, the amino acid composition of predicted proteomes (Fig. 3d), and the average carbon (C-ARSC) and nitrogen (N-ARSC) atoms in per amino acid residual chains (Fig. 3e and f) differed among clusters.

**Figure 4:**
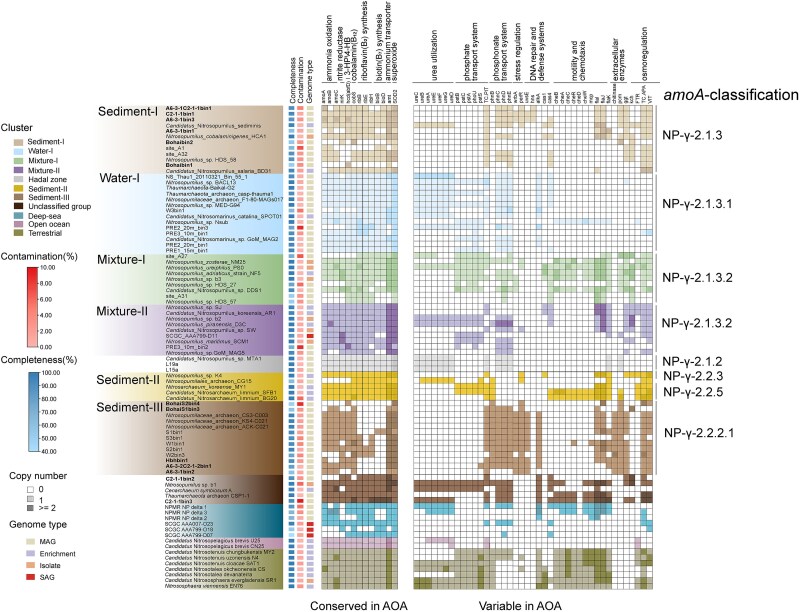
Distribution of key metabolic genes across the major *Nitrososphaeria* clusters. Different shades of color represent the presence and absence of key genes in the heatmap.

A few gene clusters were shared across all genomes with completeness >80% and contamination <5% (Supplementary Fig. S12). The pan-genome openness [51] was highest in the Mixture-I cluster, and lowest in Water-I and Sediment-III clusters (Supplementary Fig. S13), reflecting positive associations between genetic and habitat diversity. A much larger size of conserved gene clusters was observed in the Sediment-III than in the Sediment-I and Water-I clusters (Fig. 4, Supplementary Table S7 and Supplementary Fig. S14). Each cluster presented unique functional genes, which possibly enabled effective adaptation to environmental changes, promoting their ecological success and diversification (Supplementary Fig. S15). However, many genes were unannotated (45.6%–48.9%), with the ratio of genes with unknown functions being significantly higher in the Sediment-I cluster than in the Sediment-III cluster (chi-square test, *P* = .017; Supplementary Fig. S14).

The capacity for aerobic ammonia oxidation and CO_2_ fixation was detected in genomes from both coastal water and sediments. Nearly all genomes encoded the electron transport chain, including NADH-quinone oxidoreductase (complex I, *nuoABDHILMN*), succinate dehydrogenase (complex II, *sdhDCBA*), terminal oxidase (complex IV, *coxAB*), and the A-type ATPase (complex V, *atpABCDEFKI*). The absence of genes encoding cytochrome *bd* oxidases (*cydAB*), which have high oxygen affinity, suggests that these AOA are adapted to oxic surface sediments where oxygen is not a limiting factor. Consequently, their abundance sharply decreases with sediment depth as oxygen becomes depleted [52, 53].

A single copy of the *amoA* gene was present in all reconstructed genomes, except in A6-3-1C2-1-1bin1 and A6-3-1bin3 (within the Sediment-I cluster), which harbored two *amoA* copies (Fig. 4 and Supplementary Fig. S16). The second copy was likely acquired through horizontal gene transfer, despite the possibility of genomic fragmentation or assembly errors. For ammonia transport, most coastal *Nitrososphaeria* possessed two copies of *amt*, encoding high-affinity, and low-affinity ammonia transporters, respectively. Urea is another vital source of ammonia for *Nitrososphaeria* [54]. Compared to the widespread presence of urea utilization genes (*ureABC*) in the Water-I cluster, the sediment-associated clusters typically lacked urease genes (Fig. 4). Phylogenetic and genetic organization analysis indicated that these genes might have been inherited from terrestrial ancestors, possibly lost during the adaptation to sedimentary environments, and subsequently evolved into a distinct cluster in seawater (Supplementary Fig. S17). It likely represents an adaptation to the higher ammonium concentrations in coastal sediments, explaining the varied ability of *Nitrososphaeria* to utilize urea in coastal environments [55, 56].

Phosphorus (P) is another crucial inorganic nutrient for microorganisms. Microbial orthophosphate transport is usually accomplished by permease proteins encoded by a high-affinity phosphate-specific transport system (*pst*) and a low-affinity inorganic phosphate transport system (*pit*). AOA intend to use the *pst* system to cope with P limitation in oligotrophic seawater with low phosphate concentrations [9]. The Sediment-I and Sediment-III clusters lacked the *pst* system and its regulator (*phoU*), and only harbored the low-affinity *pit* system (Fig. 4). In contrast, the *pst* system was widely present in members of the water and mixture clusters, with some possessing both systems. These clusters may have acquired the *pst* system from their common marine ancestors (Supplementary Fig. S18). The higher phosphate levels in sediment settings likely reduce the need for a high-affinity transport system [9]. In addition, most coastal AOA lineages, except for the Sediment-II cluster, contained *phnCDE* genes, which are typically involved in phosphonate transport. Since these clusters lacked the key genes for degrading phosphonates and given the recently demonstrated involvement of *phnCDE* in phosphate transport [57], AOA may also use PhnCDE to transport phosphate. In contrast, both Sediment-III and Sediment-II clusters also contained *phnB*, the role of which has not been elucidated and requires further investigation.

The genetic capacity for organic matter metabolism was investigated in coastal ocean *Nitrososphaeria*. Seawater-inhabiting genomes encoded more transporters than those present in sediments (Wilcoxon test, *P* < .05, Supplementary Fig. S19), which may provide an advantage for better scavenging of the limited organic substrates available [58]. No significant differences were observed in the number of CAZymes and peptidases between sediment and water-derived genomes, except for a higher CAZyme count in Sediment-II (Supplementary Fig. S19). Additionally, no significant differences were observed in the number of peptidases across the distinct clusters (Supplementary Fig. S19). However, the capacity for chitin degradation (GH18 family of chitinases) was exclusively found in Sediment-III genomes (11 out of 15) (Fig. 4 and Supplementary Fig. S20). These chitinases contained the conserved SxGG substrate-binding site and DxxDxDxE catalytic motif [59, 60], but with a substitution of the third aspartate (D) by asparagine (N) (Supplementary Fig. S21). The chitinase sequences in Sediment-III genomes closely resemble bacterial chitinases (Supplementary Fig. S20a), suggesting a possible transfer from bacteria. Additionally, AOA belonging to the sedimentary clusters, especially the Sediment-III cluster, may need to absorb external riboflavin and biotin (Fig. 4) due to the reduced capacity for biosynthesis of these vitamins. These vitamins are key cofactors for biotin-dependent enzymes in AOA, including those involved in CO_2_ fixation [61].

Coastal sediments accumulate high concentrations of pollutants and heavy metals due to rapid urbanization and industrialization [62]. Genomes from the sediment clusters contained the *kch* gene, encoding for the voltage-gated potassium ion channel protein, the *tc.apa* gene, encoding for the alkaline amino acid/polyamine antiporter and the *vit* gene, encoding for the vacuolar iron transporter family protein (Fig. 4) that may confer resistance to heavy metals through the efflux of metal ions. Most genomes in the Sediment-III cluster and some in the Sediment-I cluster presented genes associated with oxidative stress defense, including *wrbA*, encoding for NAD(P)H dehydrogenase (quinone) that functions to prevent generation of reactive oxygen species [63], and *perR*, encoding a peroxide stress response regulator (Fig. 4). This may indicate active aerobic metabolism in the benthic lineage. Moreover, the sediment-associated cluster genomes harbored the *uvsE* gene, which encodes the UV DNA damage endonuclease (Fig. 4), potentially involved in repairing oxidative and desiccation-induced DNA lesions [64]. Thus, its presence likely reflects a broader adaptive DNA repair strategy under benthic stress conditions. Finally, genes for both flagellum biosynthesis (*flaI*, *flaJ*, *flaK*) and chemotaxis (*cheABCDRW*) were retrieved in the Mixture-I cluster genomes (Fig. 4), indicating the capacity of these microorganisms to adapt to spatially changing habitats. The genomes of the sediment clusters contained flagella-related genes but lacked chemotaxis-related genes, suggesting that these microorganisms may utilize flagella for adhesion while they likely lost the chemotaxis-driven ability to seek out nutrients [65, 66].

### Habitat transition and genetic diversity in coastal water and sediments

**Figure 5:**
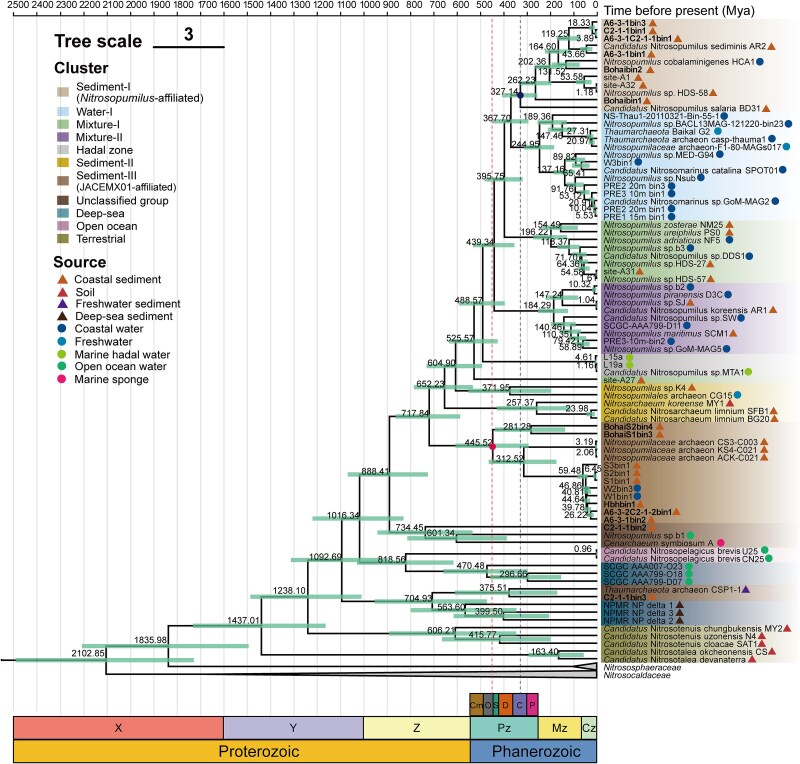
Evolutionary timeline of *Nitrososphaeria* performed by MCMCTree. The Bayesian tree was based on 122 conserved archaeal marker proteins rooted with DPANN, with the horizontal bars at each internal node representing 95% credibility intervals for divergence time estimates, and indicating the estimated divergence times of two major coastal clusters, including the JACEMX01-affiliated cluster (~446 Mya) and the *Nitrosopumilus*-affiliated cluster (~327 Mya) Cm, Cambrian; O, Ordovician; S, Silurian; D, Devonian; C, Carboniferous; P, Permian; X, Paleo-Proterozoic; Y, Meso-Proterozoic; Z, Neo-Proterozoic; Pz, Paleozoic; Mz, Mesozoic; Cz, Cenozoic; Mya, million years ago.

The complex evolutionary history of *Nitrososphaeria* residing in the coastal environments was punctuated by multiple habitat transitions. Our divergence time estimates for *Nitrososphaeria* (~2103 Mya, Fig. 5 and Supplementary Fig. S22) were highly consistent with previous archaeal dating studies [15, 22, 67]. To account for ongoing debates on archaeal rooting [43, 68–70], we tested both DPANN- and *Euryarchaeota*-rooted scenarios, which yielded nearly identical results (Supplementary Fig. S23). To maintain consistency with hypotheses that propose DPANN as a basal lineage [43], we adopted the DPANN-rooted topology within our molecular dating framework, which overall demonstrated robustness of the inferred evolutionary timeline. Phylogenetic dating suggests that the common ancestors of coastal *Nitrososphaeria* emerged ~718 million years ago (Mya) (Fig. 5 and Supplementary Fig. S22). These ancestors may have first been established in sedimentary settings and then have diversified into multiple benthic clusters, forming distinct genera. Among them, the Sediment-III cluster (JACEMX01) emerged around 446 Mya (95% highest posterior density: 292–619 Mya), placing its origin in the late Ordovician period of the Paleozoic Era. It likely represented the earliest *Nitrososphaeria* colonizer of coastal benthic environments with extant descendants. This origin occurred before the diversification of other major coastal *Nitrososphaeria* clusters (e.g. the ~327 Mya emergence of *Nitrosopumilus*-affiliated lineages) and coincided with a relatively warm paleoenvironment, in contrast to the cooler conditions of the Carboniferous period [71]. The coastal benthic *Nitrososphaeria* then evolved into planktonic-adapted clusters (e.g. the Water-I cluster), along with the expansion of their habitats into the water column. The occurrence of multiple monophyletic mixture clusters before the emergence of the Water-I cluster may reflect an ongoing process of niche partitioning, indicating several independent events of adaptive radiation from marine sediments to the water column, and promoting the diversification of *Nitrososphaeria* in coastal settings (Fig. 5 and Supplementary Fig. S22).

**Figure 6:**
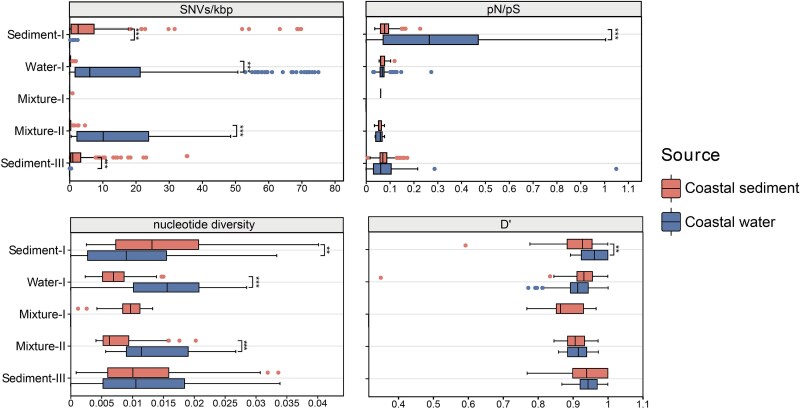
Key genome-wide evolutionary metrics of *Nitrososphaeria* lineages in coastal water and sediments, including SNV density, *pN/pS*, nucleotide diversity and D'. ^***^P < .001; ^**^P < .01; ^*^P < .05.

To gain insights into the evolutionary ecology of coastal *Nitrososphaeria*, we calculated *D*′, *pN/pS*, nucleotide diversity, and SNVs/kbp for the distinct clusters at the genomic level (Fig. 6, Supplementary Tables S8 and S9). All genomes included in the microdiversity analyses had an average coverage ≥1×, indicating robust read support for variant-based inference (Supplementary Fig. S24, Supplementary Tables S8 and S9). The SNV density (SNVs/kbp) and nucleotide diversity for both sediment- and water-derived clusters (Fig. 6) were higher in their respective environments of origin. It may indicate a better adaptation of the planktonic and benthic clusters to their specific habitats, thereby consolidating their environmental preference and niche partitioning. In addition, both groups were under the influence of purifying or stabilizing selection in their favorable environments, as indicated by low *pN/pS* values. Although the *D*′ values indicated generally low rates of homologous recombination, the Sediment-I cluster may have undergone a higher rate of homologous recombination in the sediment than in the seawater environment. A similar trend was also observed for Water-I. High homologous recombination rates may have contributed to the higher nucleotide diversity observed in the Sediment-I and Water-I cluster genomes within their respective favorable environments. Additionally, the SNV density and nucleotide diversity were higher for planktonic clusters in water compared to benthic clusters in sediments (Wilcoxon test, *P* < .01). This mirrored the intensive evolutionary diversification promoted by seawater environmental heterogeneity, which distinct levels of river runoff and human discharges may explain.

## Discussion

The coastal ocean is one of the most significant habitats for AOA on Earth [10, 24]. While *Nitrosopumilus* has been recognized as a representative group of coastal AOA [9], our findings revealed a previously uncharacterized high diversity within this functional microbial group at the genus level (Fig. 2 and Supplementary Fig. S2). Dominant genera of the coastal benthic *Nitrososphaeria* community included not only *Nitrosopumilus* but also organisms affiliated with the uncultivated JACEMX01, suggesting that this lineage plays a crucial ecological role in these environments [49]. In addition, we demonstrated that these two genera differed in relative abundance across globally distributed (spanning more than 16 000 km) coastal sediments, with latitude playing a critical role in driving niche specialization. The prevalence of JACEMX01 in sediments at lower latitudes (20–30°N) may reflect an adaptation to relatively warmer conditions. This was supported by the higher predicted optimum growth temperature of JACEMX01 compared to *Nitrosopumilus*. By contrast, members of *Nitrosopumilus* may be more tolerant to colder conditions, consistent with previous observations of its dominance in high-latitude marine environments [14]. However, in regions such as North America and northern Europe, the latitudinal transition was less pronounced, likely due to limited sediment metagenome coverage and the strong influence of local environmental factors (e.g. salinity, nutrient availability, anthropogenic impacts), as well as the mixing of nearshore and estuarine sites with distinct physicochemical conditions. These inferences are consistent with the environmental metadata collected from the 18 analyzed sediment samples (Supplementary Table S1), which showed a general trend of decreasing in situ temperature with increasing latitude.

Large within-genus diversity was observed in *Nitrosopumilus*, which aligned with previous assessments of AOA diversity based on the *amoA* [6] and 16S rRNA [47] genes. While these previous studies revealed that the majority of phylotypes within the *Nitrosopumilus* genus remain uncultivated, the coexistence patterns of different *Nitrosopumilus* phylotypes have not been defined. Here, we provided evidence that habitat heterogeneity between sediments and seawater represents an important driving force for the intra-genus diversification of *Nitrosopumilus* in the coastal ocean. Evolutionary divergence under selective pressures like habitat transition (benthic *vs.* pelagic) has been observed in groups across broad taxonomic levels (e.g. phylum and class) in both freshwater [72] and marine environments [73], as well as in planktonic and benthic AOA at the genus level from the deep sea [12]. Together with the newly obtained findings, this suggests diverse modes of AOA transitioning between marine sediments and pelagic environments, which were likely driven by distinct mechanisms such as resource availability and environmental variability (e.g. oxygen levels).

Similar to previous observations [72, 74], genome streamlining likely occurred in planktonic clusters compared to benthic ones, with associated lower genome sizes, lower GC contents, and higher coding densities (Fig. 3). The loss of genes related to motility, heavy metal resistance, and osmoregulation in the planktonic Water-I cluster of *Nitrosopumilus* indicates an adaptation to a less stressful environment in seawater. In contrast, the Water-I cluster acquired an enhanced ability to use ammonium derived from urea by the *ure* operon, to acquire phosphate via a high-affinity phosphate transport system, and to transport distinct substrate types by encoding additional membrane transporters (Fig. 4). This versatile manner of substrate and nutrient uptake for pelagic AOA was likely an adaptation to relatively nutrient-depleted conditions, given the lower concentrations of organic/inorganic nutrients (ammonium and phosphate) in coastal water compared to sediments [9]. On the other hand, the benthic clusters had an advantage in thriving under high-stress conditions, such as metal-rich and oxygen-variable environments [75], due to a higher prevalence of genes potentially involved in stress regulation, DNA repair, and heavy metal resistance. This advantage appeared to be more pronounced in the Sediment-III cluster (JACEMX01) compared to the Sediment-I cluster of *Nitrosopumilus*, suggesting distinct metabolic strategies between these two benthic lineages. Additionally, these lineages differed in their heterotrophic potential, particularly in the specific capacity of the Sediment-III cluster to utilize chitin, likely due to the horizontal transfer of genes from bacteria. However, the enzymatic activity of these genes requires further confirmation. This metabolic specialization may be attributed to differences in their frequency of contact with substrates in their respective favorable environments. While additional studies are necessary to confirm these findings, our analysis identified distinct lineages of AOA specialized in marine sediments that differed in their genetic and physiological characteristics, not limited to favorable growth temperatures and substrate spectra. The high metabolic diversity of *Nitrososphaeria* in coastal zones suggests that the underexplored metabolic potential of these archaea in the coastal ocean is substantial.

It has been proposed that coastal *Nitrososphaeria* originate from ancestral lineages in terrestrial environments [15, 76]. These terrestrial lineages evolved into coastal-specific groups upon entering marine settings [15]. The inferred timeline of transition in this study was comparable to previous estimates for the formation of coastal clusters, which were represented mainly by *Nitrosopumilus* and Ca. Nitrosoarchaeum [15, 67]. However, we demonstrated that Sediment-III, which evolved before the distinct clusters of *Nitrosopumilus*, was likely a more ancient cluster of AOA in coastal areas. Given the higher abundance of Sediment-III in sediments compared to the water column, we hypothesized that the ancestor of this cluster may have been transported from terrestrial environments to coastal sediments by attaching to particles, as indicated by the presence of flagellum-related genes. Our reanalysis of previously reported amplicon data in the Bohai Sea water and sediments [77] supported this hypothesis, revealing that the particle-associated and benthic *Nitrososphaeria* communities share the same dominant species, which has a low abundance in the free-living community (Supplementary Fig. S25). It suggests that surface sediments were likely the first environment where the coastal *Nitrososphaeria* community began to establish. The consistent occurrence of such transport events may have facilitated independent evolution into phylogenetically distinct lineages in coastal sediments. Because sediment resuspension driven by hydrodynamic forcing occurs naturally and recurrently in marginal seas [78], benthic AOA that were repeatedly dispersed into the water column may have gradually evolved into specific water-preferred clusters. It was notable that the water-derived genomes were scattered into different phylogenetic clusters. This may have resulted from the parallel migration of distinct clusters from coastal sediments to the overlying waters. The observation of phylogenetically separated benthic and pelagic clusters within *Nitrosopumilus*, along with their low *pN/pS* values, suggests that they may have each reached an adaptive mediated optimum in their respective environments following niche separation. After thriving in these environments, they may have begun to diversify to maintain intrapopulation diversity through a low rate of homologous recombination. By contrast, the unresolved phylogeny of water and sediment-derived genomes in the Mixture clusters suggests ongoing processes of niche separation between benthic and pelagic environments.

Our study provided a detailed insight into the diversity, biogeography, and evolution of the globally dominant archaeal lineages in coastal oceans. The co-dominance of two phylogenetically distinct clusters at the genus level unveiled a highly diverse community of *Nitrososphaeria* across global marginal sea sediments. The varying latitudinal patterns, physiological potentials, and metabolic strategies among these benthic lineages provided evidence of parallel and independent evolutionary radiation and adaptation to sediment settings. The Sediment-III cluster (JACEMX01) likely originated earlier than *Nitrosopumilus* as an early colonizer of the coastal *Nitrososphaeria* community, with sediments probably offering the initial niche for its development. Sediment resuspension facilitated cell dispersal into the overlying water column and promoted subsequent adaptive divergence due to habitat transition. This process probably occurred at different stages for different lineages. These findings also open new avenues of research, including the factors underpinning the differential diversification and coexistence of the benthic *Nitrososphaeria* clusters, as well as the relative contribution of the different clusters to the carbon and nitrogen cycles in the coastal ocean.

## Supplementary Material

Supplementary_materials_ycaf234

## Data Availability

Metagenome sequences (PRJNA1159446) and MAGs (SAMN43765377~SAMN43765389) from this study are available on the NCBI database.
