# Supplementary material for: Purifying selection and low recombination facilitated sequential colonization of benthic and pelagic coastal ocean by ammonia-oxidizing archaea
Source: ISME Commun. 2025 Dec 8;5(1):ycaf234. doi: 10.1093/ismeco/ycaf234 (PMC12753310; doi:10.1093/ismeco/ycaf234)
Supplement: Supplementary_materials_ycaf234 [file supplementary_materials_ycaf234.zip › 03_Supplementary_information.docx]

**Supplementary information**

**Purifying selection and low recombination facilitated sequential colonization of benthic and pelagic coastal ocean by ammonia-oxidizing archaea**

Gaoyang Ren^1,2,3^, Cécile Gubry-Rangin^4^, Wenhao Wang^1,2,3^, Ronghua Liu^1,2,3^, Jiao Liu^1,2,3^, Jinmei Liu^1,2,3^, Xiao-Hua Zhang^1,2,3^, Jiwen Liu^1,2,3^*

**Material and methods**

**DNA extraction**

Briefly, 5 g of sediment samples were ground in liquid nitrogen. Subsequently, 16.5 mL of extraction buffer was added, which consisted of 100 mM EDTA (pH 8.0), 100 mM Tris-HCl (pH 8.0), 1.5 M NaCl, 100 mM sodium phosphate (pH 8.0), and 10% CTAB. Protease K and SDS were then sequentially introduced, followed by gently mixing. The mixture was incubated first at 37°C, then at 65°C, and subsequently centrifuged at 6000×g for 20 minutes at room temperature. The supernatant was subjected to phenol-chloroform extraction. DNA was precipitated by adding 0.6 times the volume of isopropanol, incubating at room temperature for 2 - 3 hours, followed by washing with 70% ethanol and centrifugation. After air-drying, DNA was dissolved in TE buffer and further purified using the DNeasy PowerSoil Kit (QIAGEN). The concentration and integrity of DNA were assessed using a NanoDrop spectrophotometer (ND - 2000; Thermo Fisher Science) and agarose gel electrophoresis.

**Phylogenetic inferences**

The AOA phylogeny was inferred from the 13 reconstructed MAGs in this study alongside 72 publicly available reference genomes (Supplementary Table S5) from diverse habitats, with a focus on coastal environments. Phylogenomic analysis was conducted using a concatenated alignment of 54 archaeal ribosomal proteins (Supplementary Table S6), following the marker set established by Seitz et al. [1] for archaeal phylogenetic reconstruction [2]. The original set included 56 ribosomal proteins; however, two markers (arCOG04167 [COG0093, L14] and arCOG04175 [COG2157, L20A]) were frequently missing across our MAGs and were excluded to maximize genome representation. Ribosomal proteins were annotated using eggNOG-mapper (v2.1.12). Individual protein sequences were aligned using MAFFT (v7.471), and poorly aligned regions were trimmed using trimAL (v1.4.rev15) [3] with default parameters. The final concatenated alignment was used for maximum likelihood tree construction. The phylogeny was constructed using two different methods, maximum likelihood and Bayesian inference. Additionally, a maximum-likelihood phylogenetic tree was inferred based on 122 conserved single-copy archaeal marker genes from the GTDB database. The maximum-likelihood tree was constructed using IQ-TREE (v2.2.2.6) [4], with the optimal substitution model for each gene family automatically determined by ModelFinder [5]. The Bayesian inference tree was built using MrBayes (v3.2.7) (average standard deviation of split frequencies <0.01) [6]. To assess the consistency of phylogenetic relationships, a strict consensus tree was generated using IQ-TREE (v2.2.2.6) with the -con 100 option. This analysis combined all phylogenetic trees from maximum likelihood and Bayesian methods, retaining only branches present in all input trees. The resulting consensus tree displayed consistent clustering patterns and relative positions of key groups.

Sequences of the 16S rRNA gene were extracted from the coastal sediment metagenomes of this study using Metaxa2 (v2.2.3) [7] and annotated against the Silva v138 database [8]. All extracted sequences were imported into QIIME 2 (v2023.7.0) [9] for clustering analysis at a 97% similarity. Representative sequences from *Nitrososphaeria* were aligned with those from the publicly available reference genomes mentioned earlier for comparison. Also, ribosomal sequences were extracted from MAGs using Barrnap (v0.9; https://github.com/tseemann/barrnap). Alignment of the 16S rRNA gene sequences derived from MAGs recovered in this study (Supplementary Table S5) was performed using MAFFT (v7.471) [10], and a maximum-likelihood phylogenetic tree was computed with IQ-TREE (v2.2.2.6) [4] using 1000 bootstrap replicates. AOA sequences were classified by linking the taxonomy of the 16S rRNA gene and the *amoA* gene, following the framework established by Wang et al. [11]. The phylogeny of key functional genes (e.g., *amoA*, *ureC*, *pstB*, and the gene encoding chitinase) was inferred using homologous sequences from the NCBI and UniProtKB databases. Sequences were aligned with MAFFT (v7.471), and trimmed with trimAL (v1.4.rev15). Phylogenetic trees were constructed in IQ-TREE (v2.2.2.6) using the maximum-likelihood method with best-fit models and 1000 ultrafast bootstraps. The resulting trees were visualized and refined in iTOL (v6).

**Genome functional annotation and metabolic analysis**

Genes predicted by Prokka (v1.14.6; default parameters) were annotated in the KEGG server (GhostKOALA) [12]. The KO orthologs were assigned to coding sequences using GhostKOALA (v2.0) with the “genus_prokaryotes + family_eukaryotes + viruses” option (Supplementary Table S7). Carbohydrate active enzymes (CAZymes; v12) were searched against dbCAN3 with default settings; only matches annotated by at least two tools were considered [13]. Peptidases were identified using BLASTp (v2.5.0+) against the MEROPS database (v12.4), selecting matches with ≥ 40% identity, > 30% coverage and an evalue < 10^-10^. Transporters were searched similarly against the Transporter Classification Database (TCDB; v2023.9.30) [14]. Genes and multiple sequence alignments (MSA) were visualized with SnapGene (v6.0.2) and GeneDoc (v2.7), respectively.


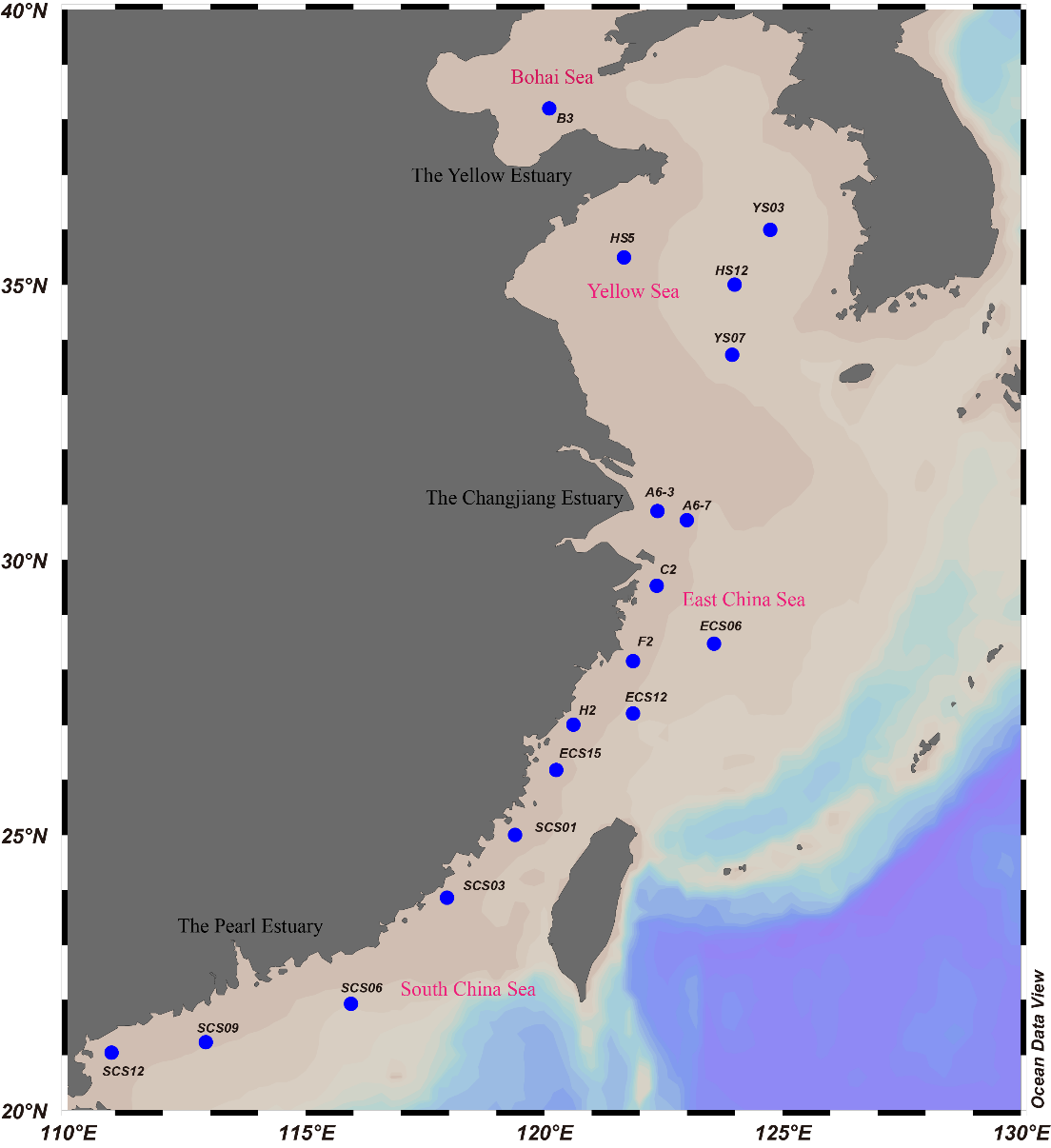


**Figure S1** Geographic map showing the sampling sites.


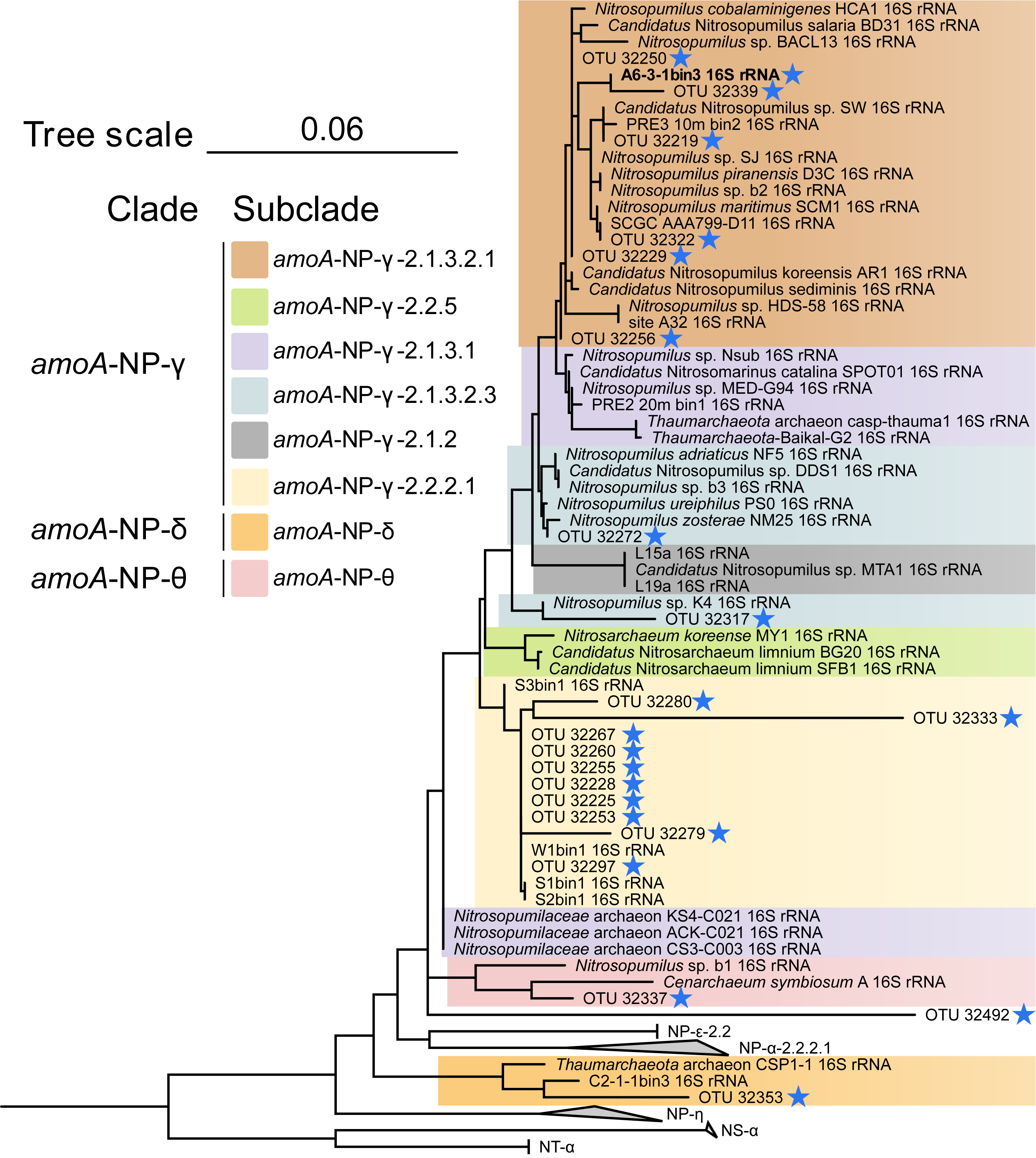


**Figure S2** A maximum-likelihood phylogenetic tree based on 16S rRNA genes extracted from *Nitrososphaeria* MAGs and metagenomic data (labeled with blue star). The tree was constructed using the GTR+F+I+G4 model. Sequences generated in this study are shown in bold.


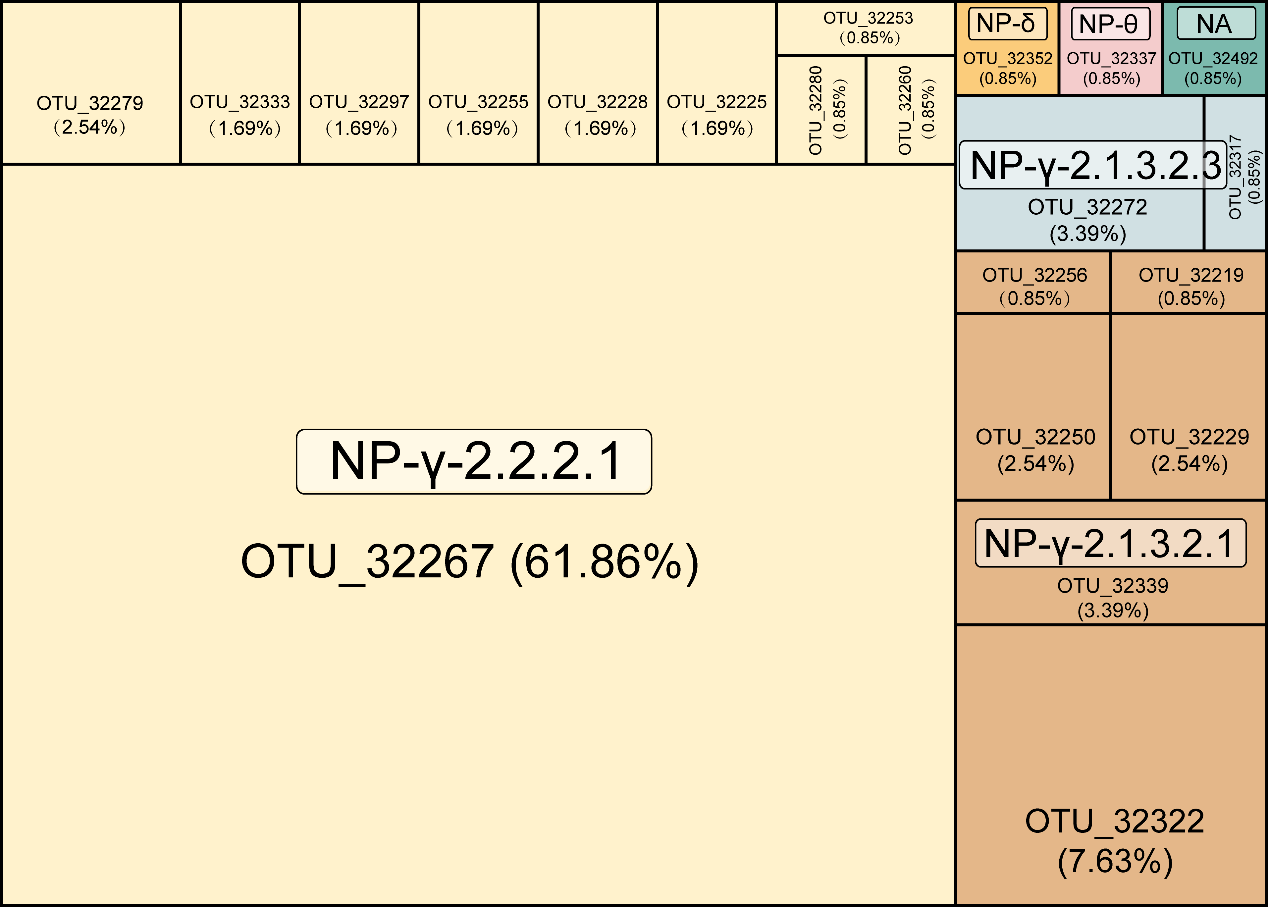


**Figure S3** Relative abundance of *Nitrososphaeria* 16S rRNA gene OTUs extracted from metagenomes*.* Each box represents an OTU, with its size and label proportional to relative abundance among all *Nitrososphaeria*-affiliated 16S rRNA gene reads. Percentages were calculated as the number of reads assigned to each OTU divided by the total number of *Nitrososphaeria* 16S rRNA reads (n = 118 reads across all samples).
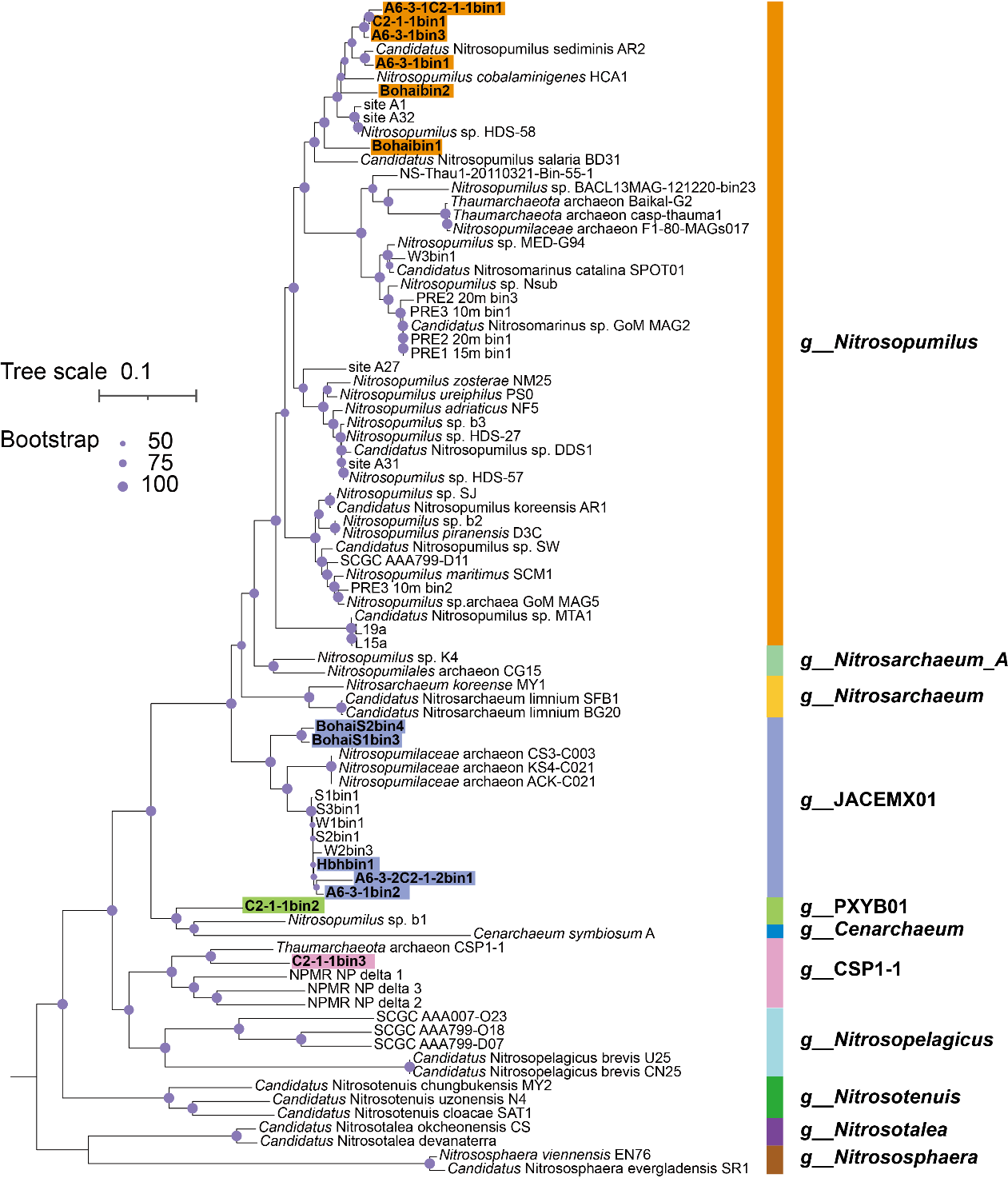


**Figure S4** A maximum-likelihood phylogenomic tree based on 54 ribosomal proteins predicted from *Nitrososphaeria* MAGs (shaded with color) and reference genomes. The tree was built using IQ-TREE (v2.2.2.6) with the LG+F+R5 model and 1000 bootstrap replicates. Taxonomic classification of each genome was labeled at the genus level based on the GTDB database.


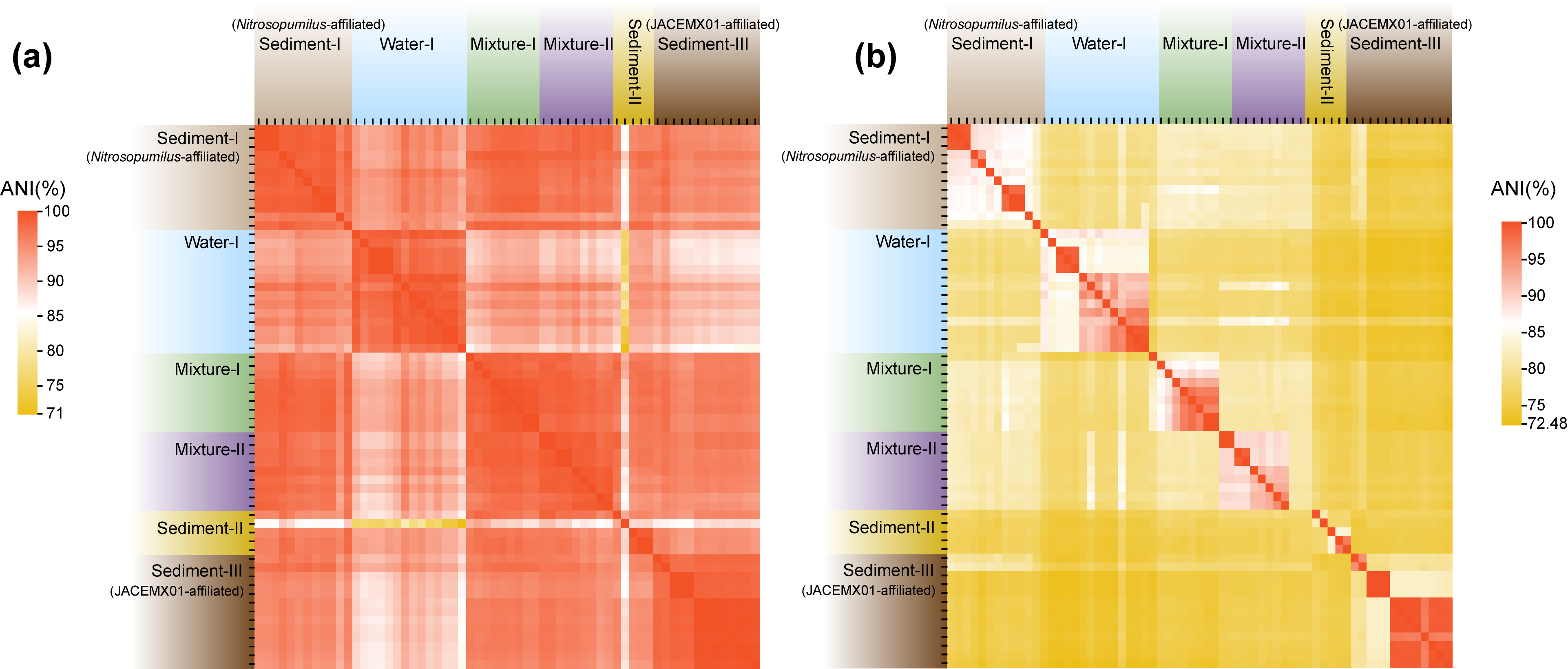


**Figure S5** The genome-wide average nucleotide identity (ANI, a) and amino acid identity (AAI, b) among coastal-derived *Nitrososphaeria* genomes. Genomes were grouped into genera based on AAI (<75%) and ANI (<90%) thresholds commonly used for genus-level differentiation.


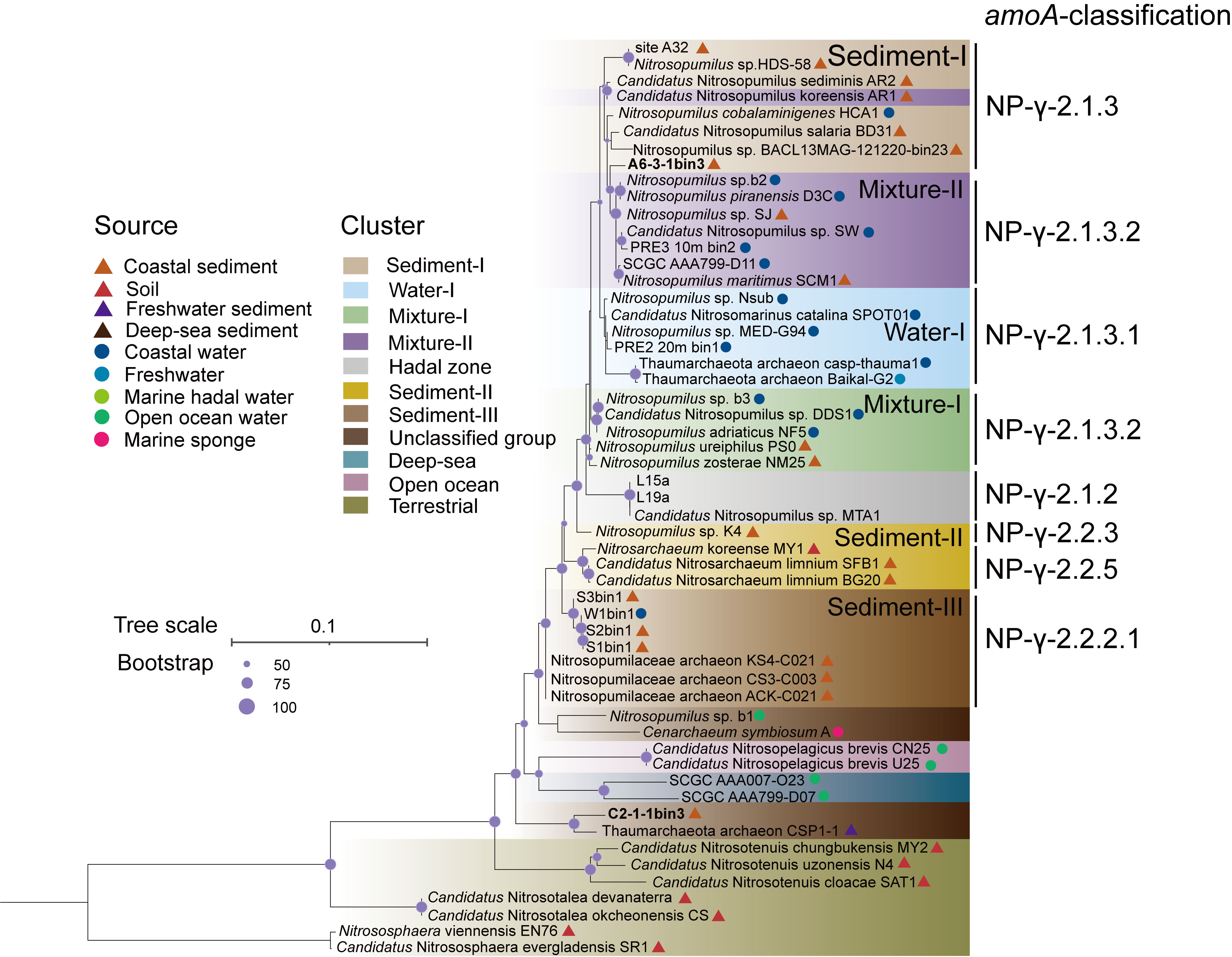


**Figure S6** The phylogeny of *Nitrososphaeria* 16S rRNA genes extracted from MAGs. The tree was built using IQ-TREE (v2.2.2.6) with the TIM3e+I+G4 model and 1000 bootstrap replicates. A6-3-1bin3 generated in this study is shown in bold. Most genomes within the Water cluster were retrieved from coastal water, while most genomes originated from coastal sediments in the Sediment cluster. In contrast, the Mixture cluster contained genomes from both coastal water and sediments.


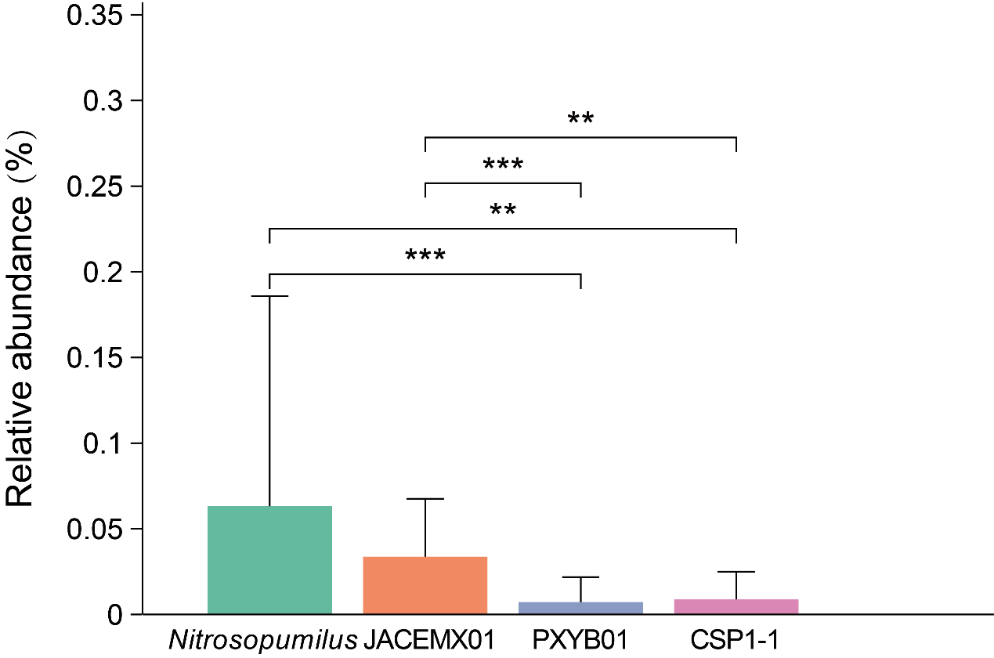


**Figure S7** Relative abundance of MAGs affiliated with different *Nitrososphaeria* genera in sediments of the Chinese marginal seas. Statistical differences were assessed using Wilcoxon rank-sum tests (****P* < 0.001; ***P* < 0.01).


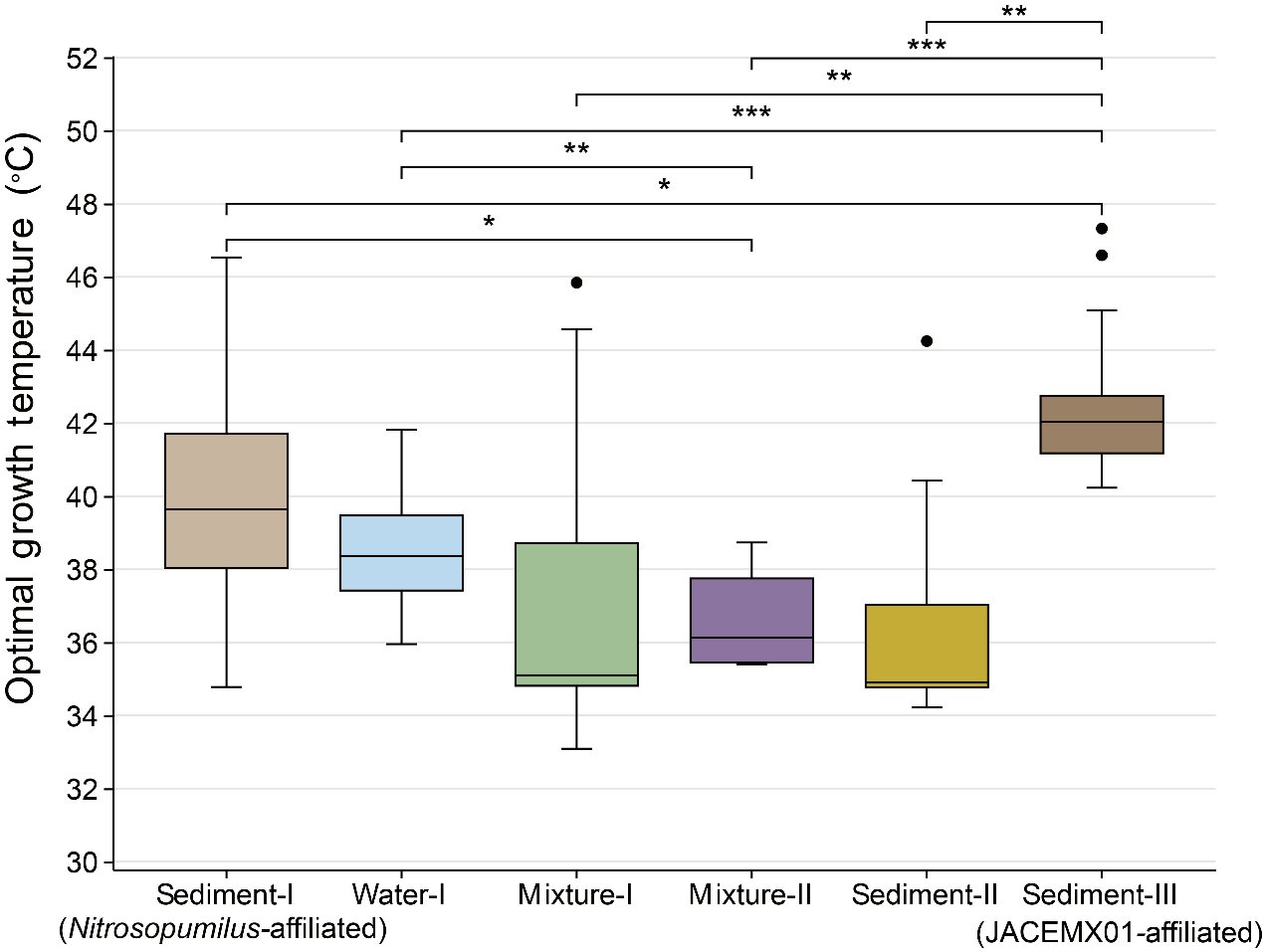


**Figure S8** Predicted optimal growth temperature in different clusters of coastal *Nitrososphaeria*. Statistical differences were assessed using Wilcoxon rank-sum tests (****P* < 0.001; ***P* < 0.01; **P* < 0.05).


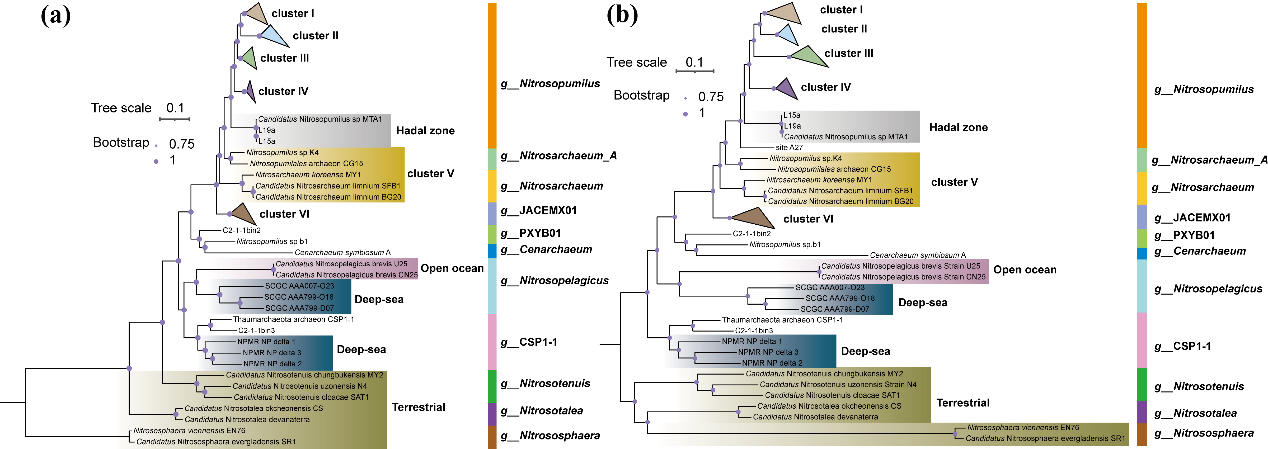


**Figure S9** The phylogeny of *Nitrososphaeria* inferred from different gene sets and algorithms. (a): A Bayesian tree inferred from 54 ribosomal proteins. (b): A maximum-likelihood tree inferred from 122 single-copy archaeal markers with the LG+F+R6 model and 1000 bootstrap replicates. Bootstrap values are indicated on nodes.


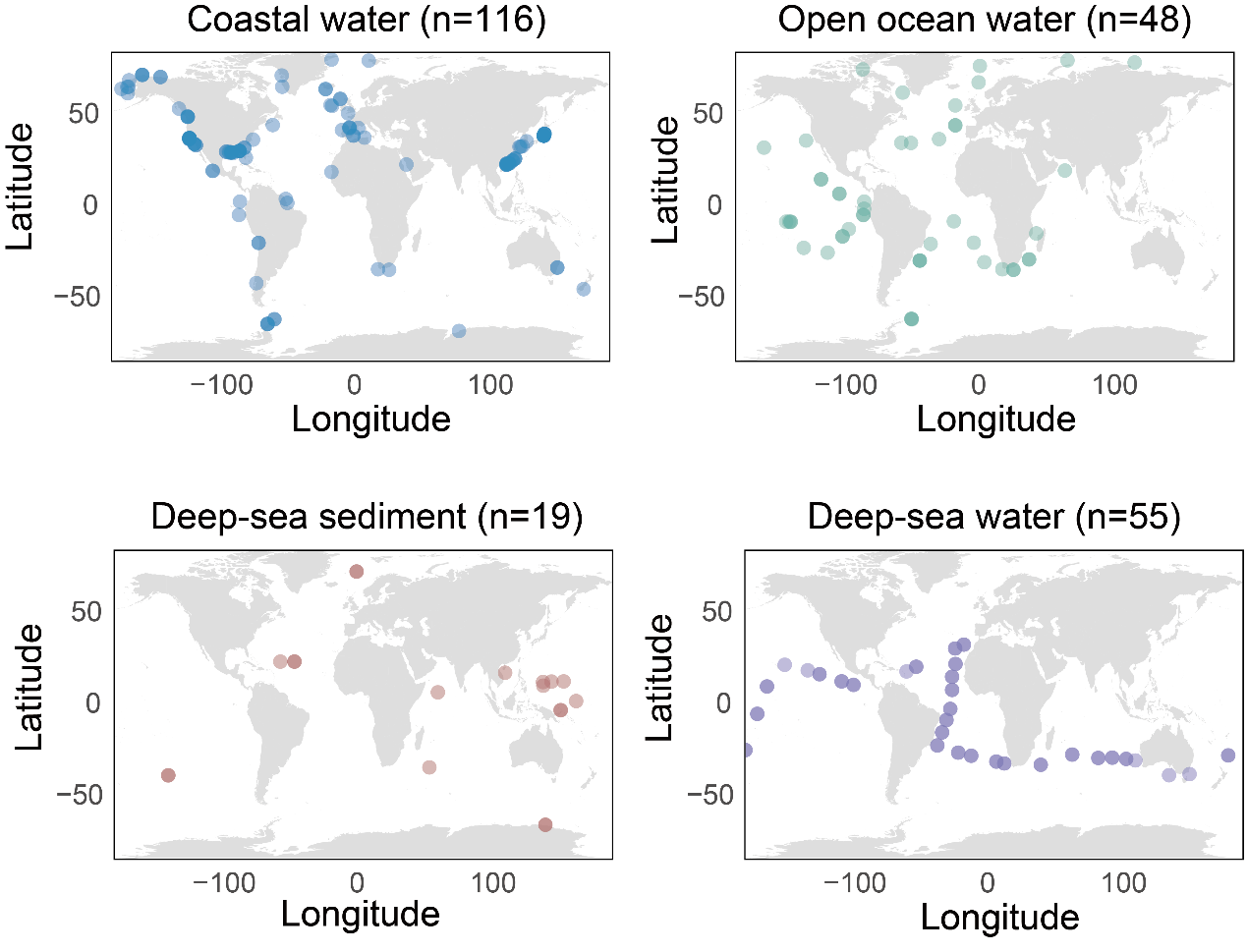


**Figure S10** Distribution of metagenomic samples retrieved from coastal water, open ocean water, deep-sea sediments and deep-sea water.


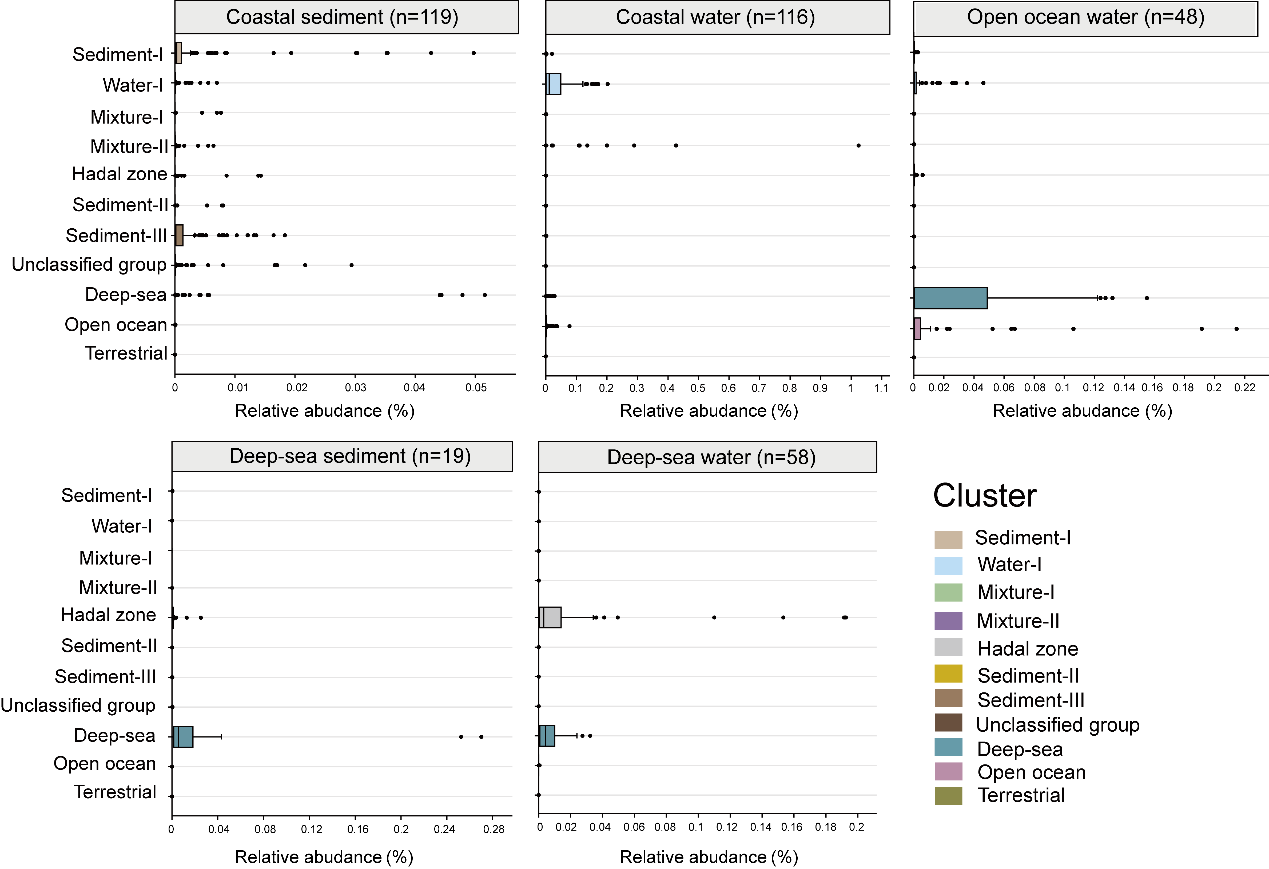


**Figure S11** Relative abundance of *Nitrososphaeria*-associated clusters in different ecological habitats, including coastal sediments, coastal water, open ocean water, deep-sea sediments, and deep-sea waters.
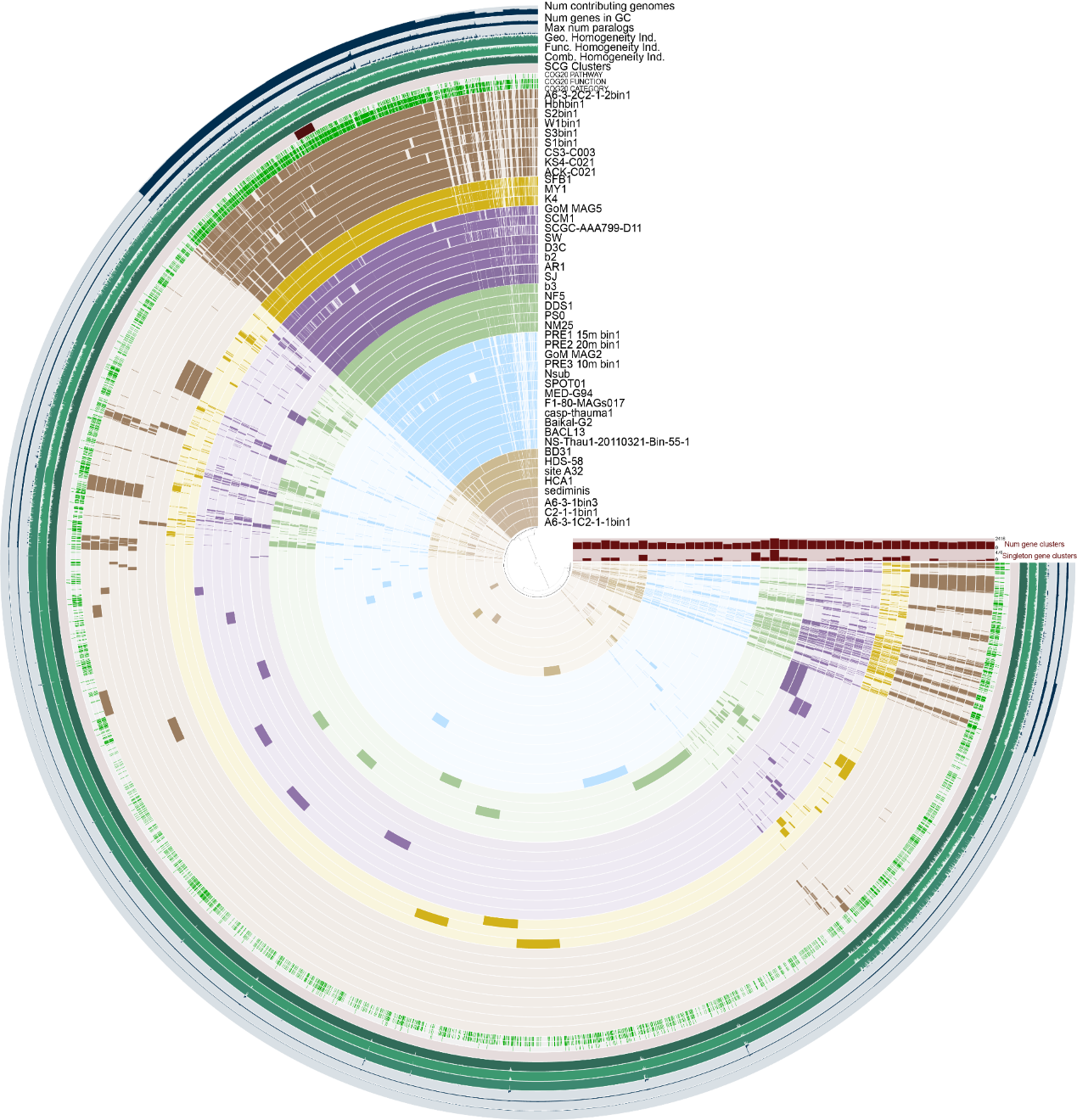


**Figure S12** Pan-genome analysis of *Nitrososphaeria*. SCG Clusters indicates the COGs cluster of single-copy conserved genes shared by all genomes. Gene clusters were ordered based on hierarchical clustering of their presence/absence (inner dendrogram).


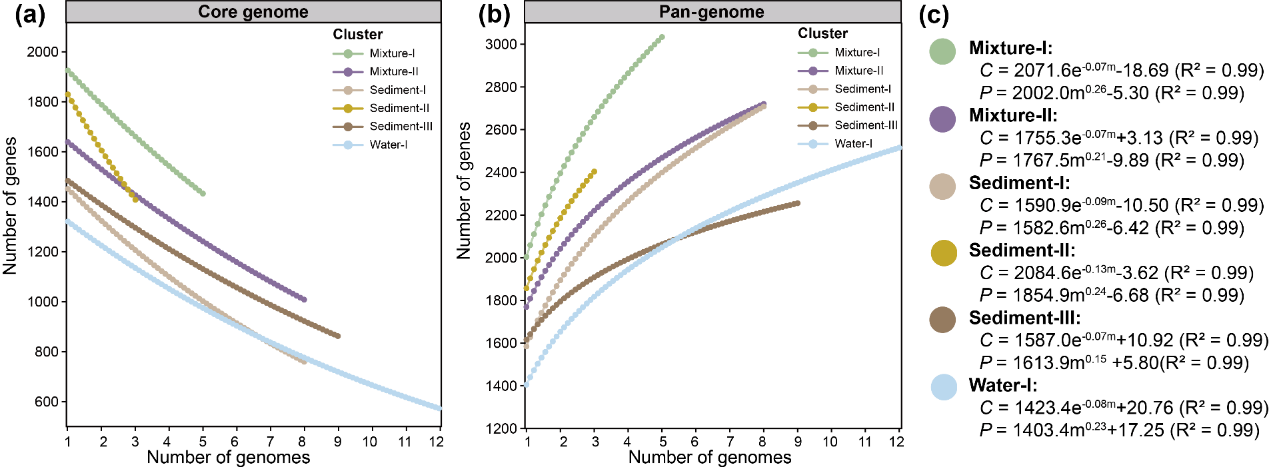


**Figure S13** The sizes of core genome (a) and pan-genome (b) of the distinct clusters. Genomes with completeness >80% and contamination <5% were used for calculation. The core genome and pan-genomes were fitted using Heaps’law (c), where *C* denotes the number of genes in core genome, *P* denotes the number of genes in pan-genome, and m denotes the number of genomes. The R² value reflects the goodness of fit of the Heap's law model.


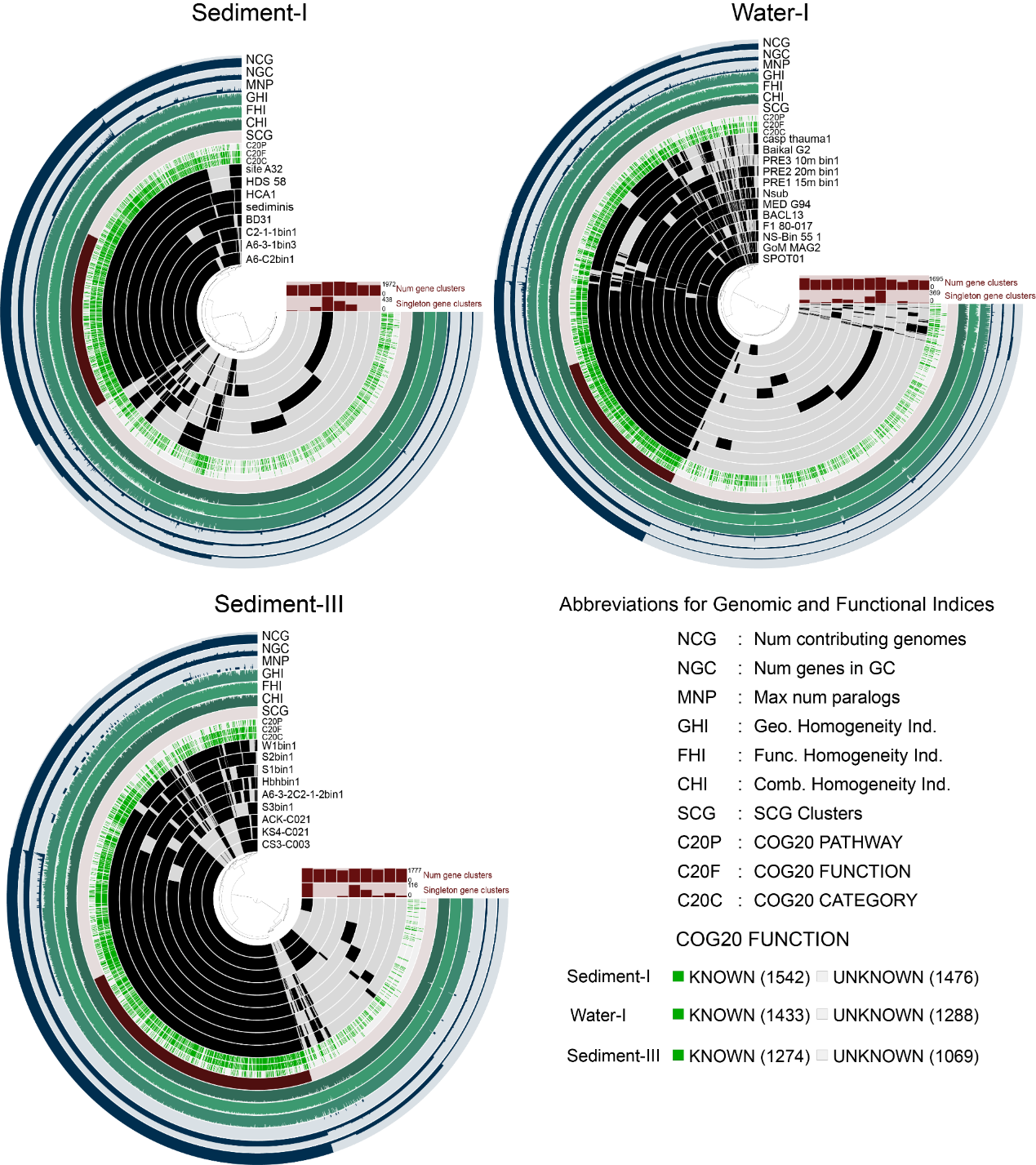


**Figure S14** Pan-genome analysis of Sediment-I, Water-I and Sediment-III clusters. The circle labeled COG20 illustrates the functions in the genomes, with known functions shown in green and unknown genes in white. The brown circular bar represents the cluster of COGs for the single conserved genes (SCGs) shared by all genomes. Inner rings indicate the presence (black) and absence (grey) of gene clusters in each genome.


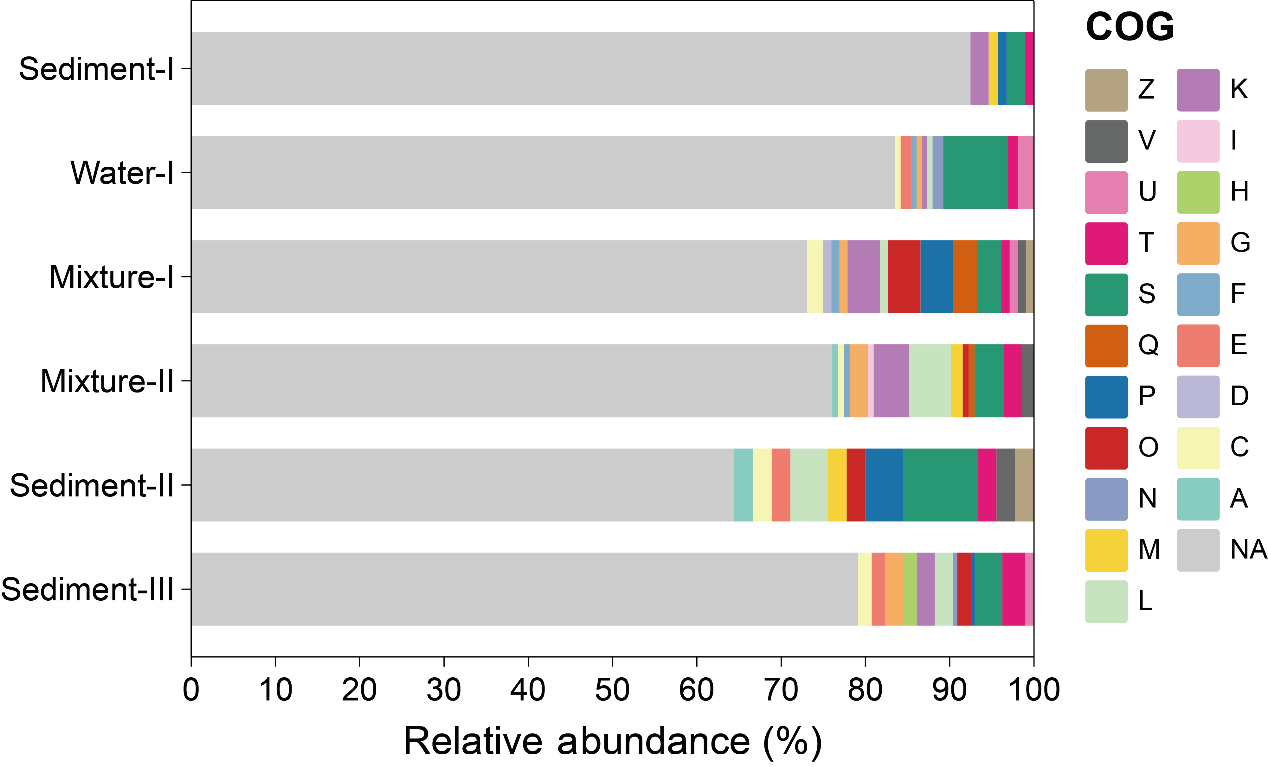


**Figure S15** The unique functional genes in each cluster. The chart shows the relative abundance of COG categories of unique functional genes across different clusters.


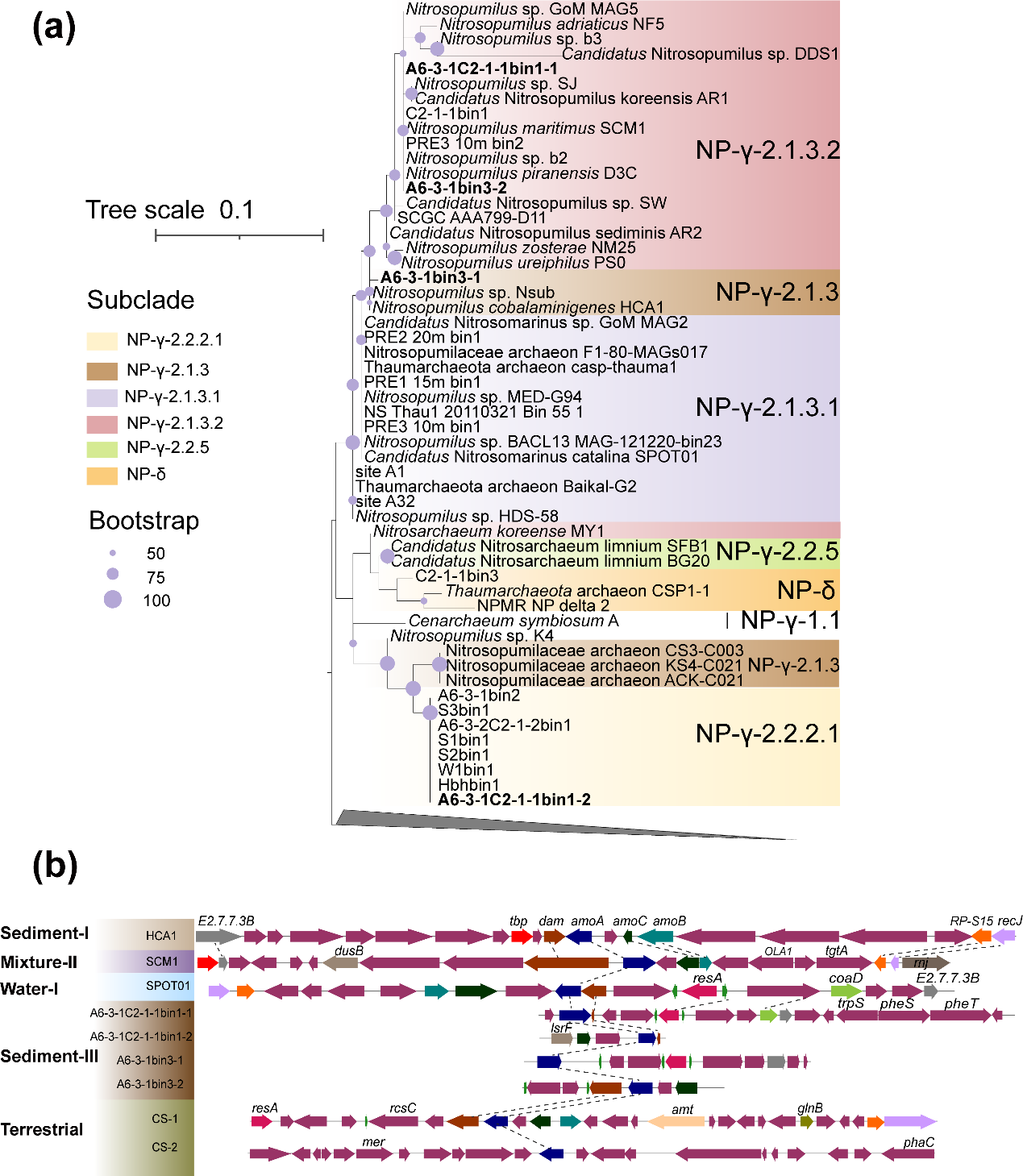
­­

**Figure S16** Phylogenetic analysis of AmoA (a) and representation of the *amoABC* gene cluster (b). (a) The tree was constructed using IQ-TREE (v2.2.2.6) with the mtZOA+G4 model and 1000 bootstraps. Sequences generated in this study are shown in bold. (b) Navy blue represents *amoA* genes, teal indicates *amoB* genes, dark green corresponds to *amoC* genes, and burgundy denotes genes of undefined functions. The functions of other colors have been labeled accordingly. Most *amoA* sequences are full-length (250–275 amino acids); a few shorter sequences were identified and found at contig edges, suggesting they are likely the result of assembly breaks rather than annotation artifacts.


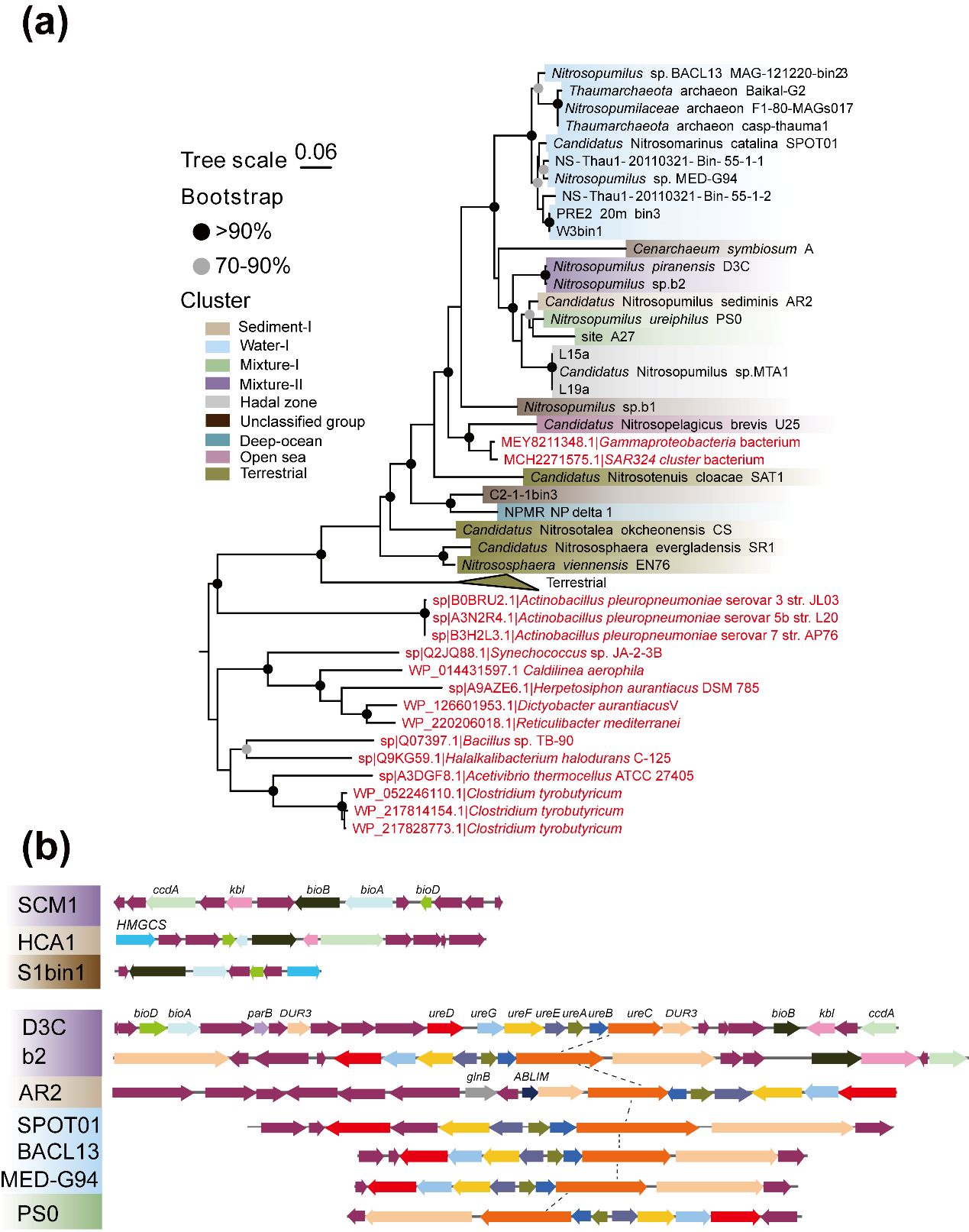


**Figure S17** Phylogenetic analysis of UreC (a) and representation of the urea gene utilization cluster (b). (a) The tree was constructed using IQ-TREE (v2.2.2.6) with model LG+I+G4 and 1000 bootstraps. Reference sequences are marked in red. (b) Red represents *ureD* genes, light blue indicates *ureG* genes, yellow corresponds to *ureF* genes, purple signifies *ureE* genes, olive green denotes *ureA* genes, dark blue indicates *ureB* genes, and orange represents *ureC* genes. Burgundy indicates genes of undefined functions.

The functions of other colors have been labeled accordingly.


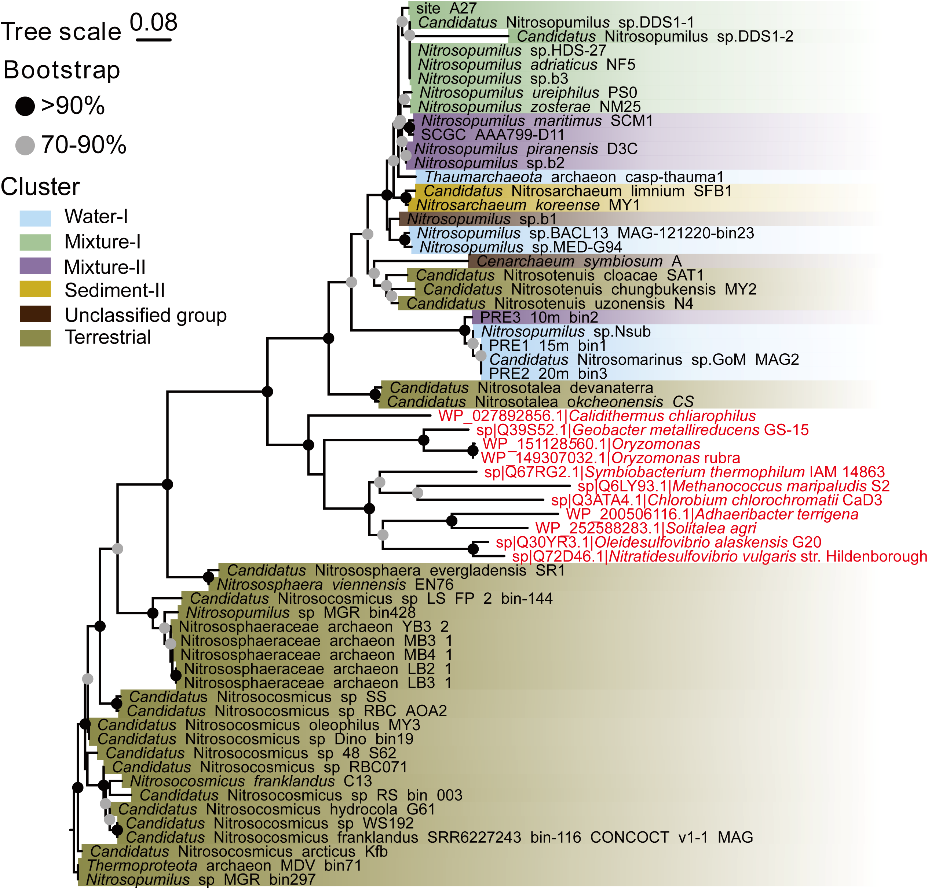


**Figure S18** Phylogenetic tree of PstB. The tree was constructed using IQ-TREE (v2.2.2.6) and model Q.yeast+I+R3 with 1000 bootstraps. Reference sequences are marked in red.


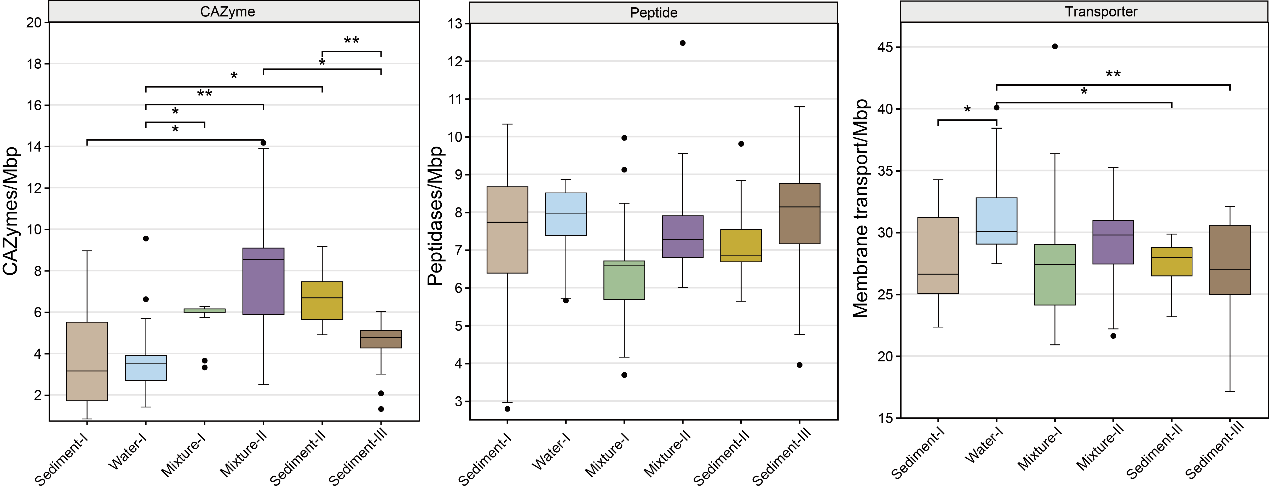


**Figure S19** Comparison of predicted carbohydrate-active enzymes (CAZymes), peptides, and transporters across genomes. Statistical differences were assessed using Wilcoxon rank-sum tests (***P* < 0.01; **P* < 0.05).


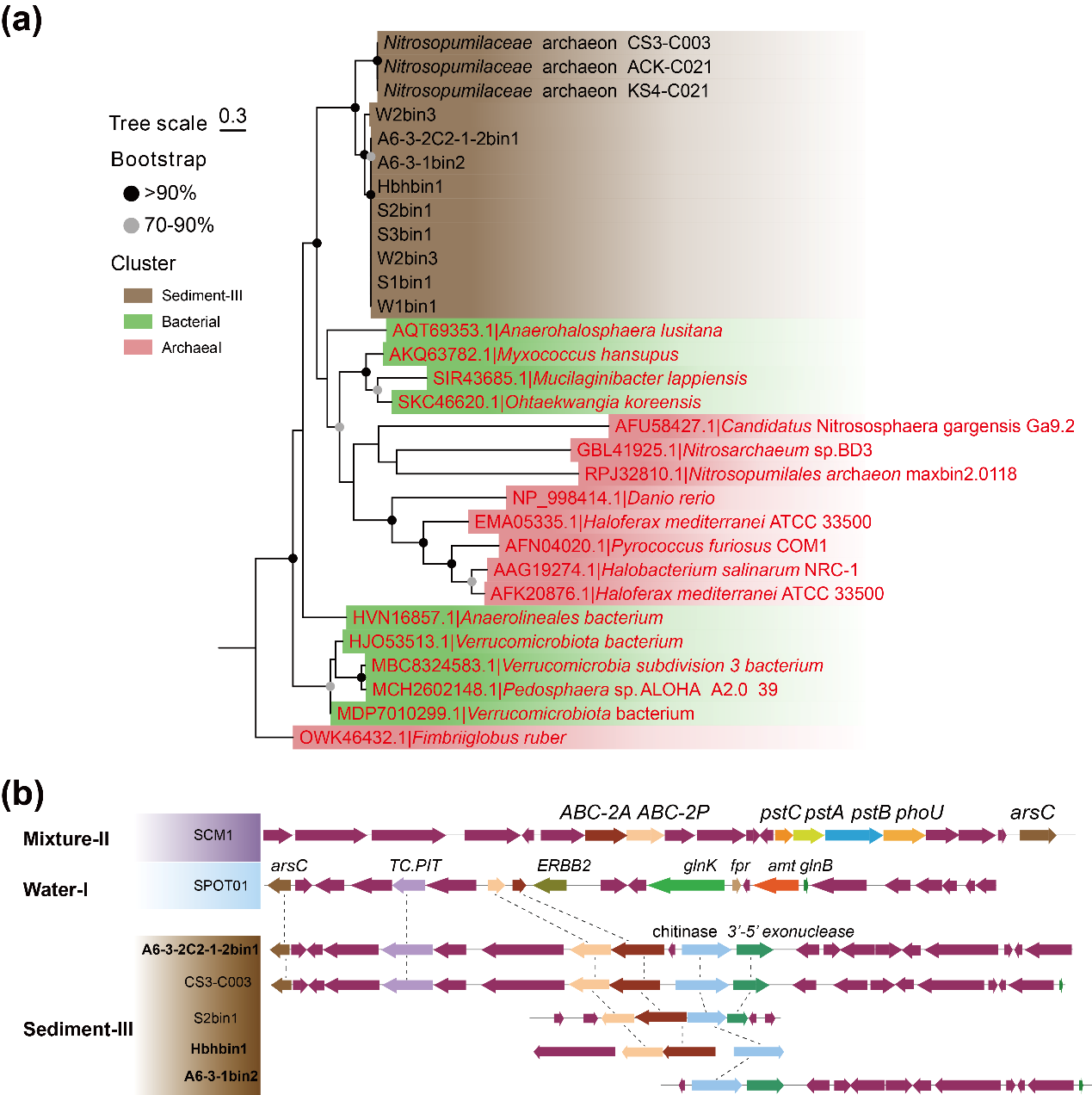


**Figure S20** Phylogenetic tree of chitinase [E3.2.1.14] (a) and representation of chitinase cluster (b). (a) The tree was constructed using IQ-TREE (v2.2.2.6) and model Q.pfam+I+G4 with 1000 bootstraps. Reference sequences are marked in red. (b) Light blue represents chitinase genes. Burgundy genes are undefined functions. The functions of other colors have been labeled accordingly.


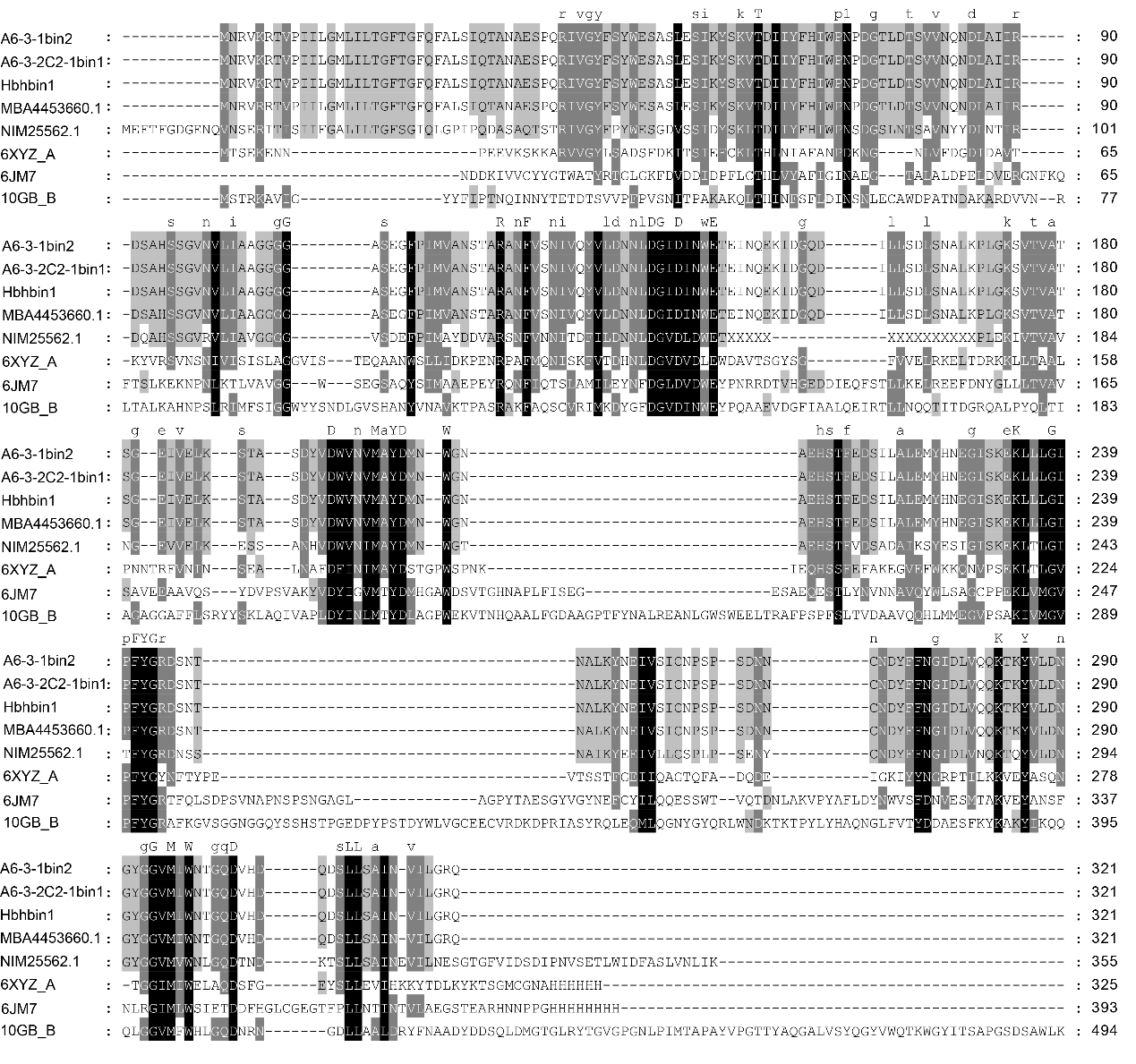
­­

**Figure S21** Multiple sequence alignment of amino acid sequences of chitinase. MBA4453660.1 is the chitinase sequence of the W2bin3. NIM25562.1 is the chitinase sequence of the *Nitrosopumilaceae*-archaeon-KS4-C021. 6XYZ_A (*Flavobacterium johnsoniae* UW101), 6JM7 (*Ostrinia furnacalis*) and 10GB_B (*Serratia marcescens*) have been shown to maintain significant activity in previous studies.


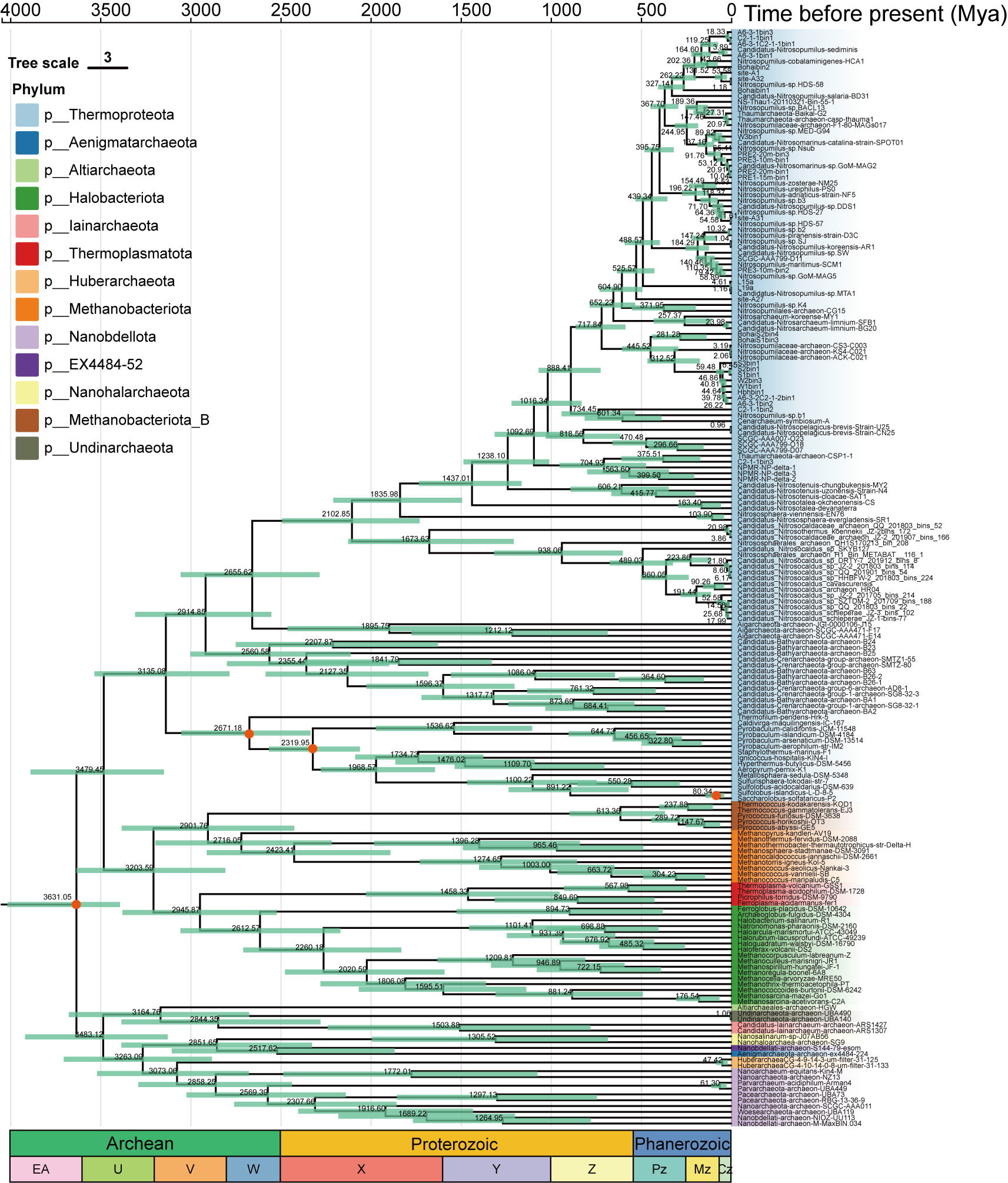


**Figure S22** Evolutionary timeline of *Nitrososphaeria* performed by MCMCTree. The Bayesian tree based on 122 conserved archaeal marker proteins was rooted with DPANN. The green horizontal bars at each internal node represent 95% credibility intervals for divergence time estimates. Nodes shown in orange-red represent the four lineages constrained in the phylogenetic analysis. EA, Eoarchean; U, Paleoarchean; V, Mesoarchean; W, Neoarchean; X, Paleo-Proterozoic; Y, Meso-Proterozoic; Z, Neo-Proterozoic; Pz, Palaeozoic; Mz, Mesozoic; Cz, Cenozoic; Mya, million years ago.


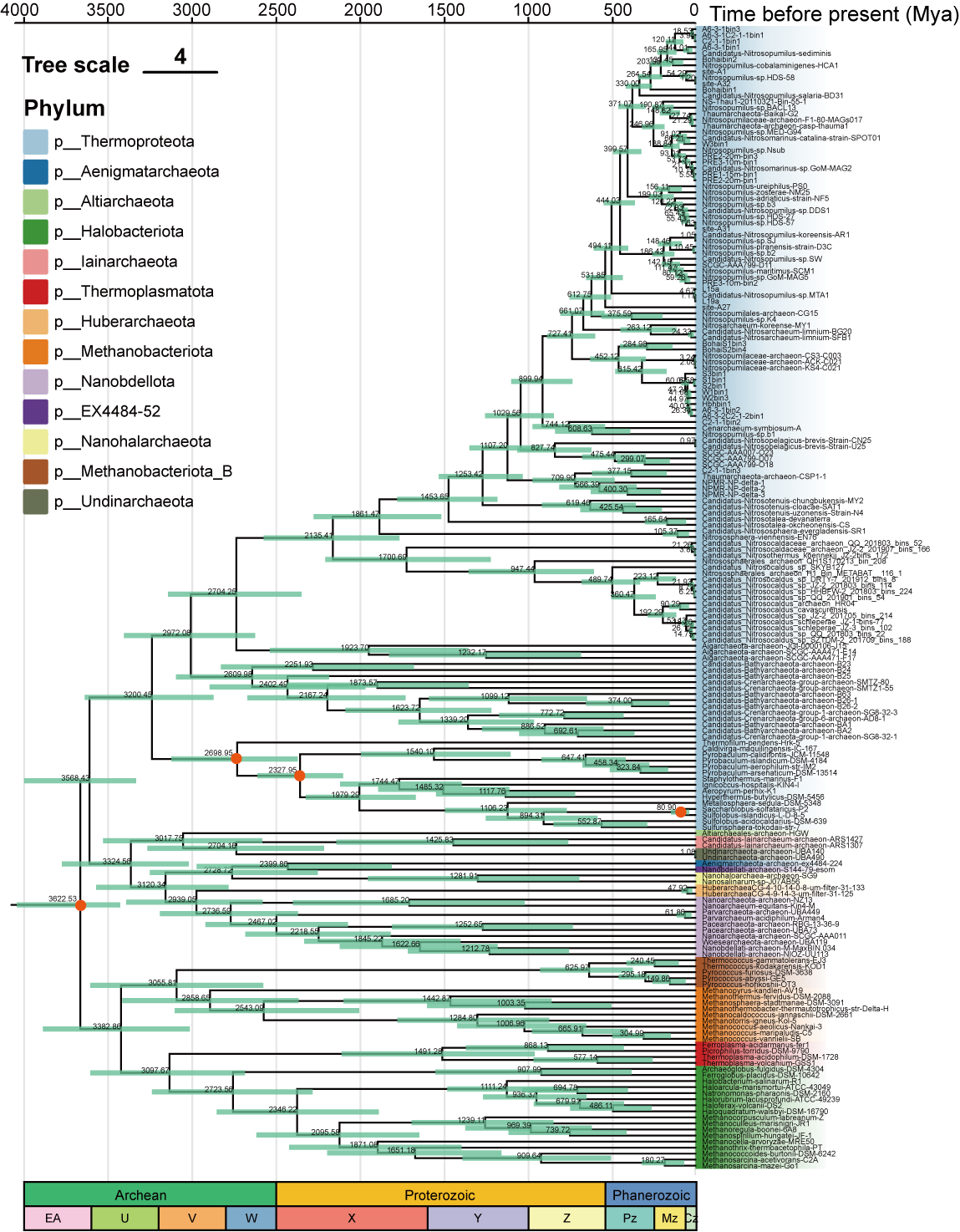


**Figure S23** Evolutionary timeline of *Nitrososphaeria* inferred with MCMCTree under an alternative rooting. The Bayesian chronogram was reconstructed from 122 conserved archaeal marker proteins but rooted with *Euryarchaeota* (non-DPANN root). Green horizontal bars denote 95% credibility intervals for divergence time estimates, and orange-red nodes represent the four calibration points used in the phylogenetic analysis. Geological time abbreviations as in Figure S22.

**
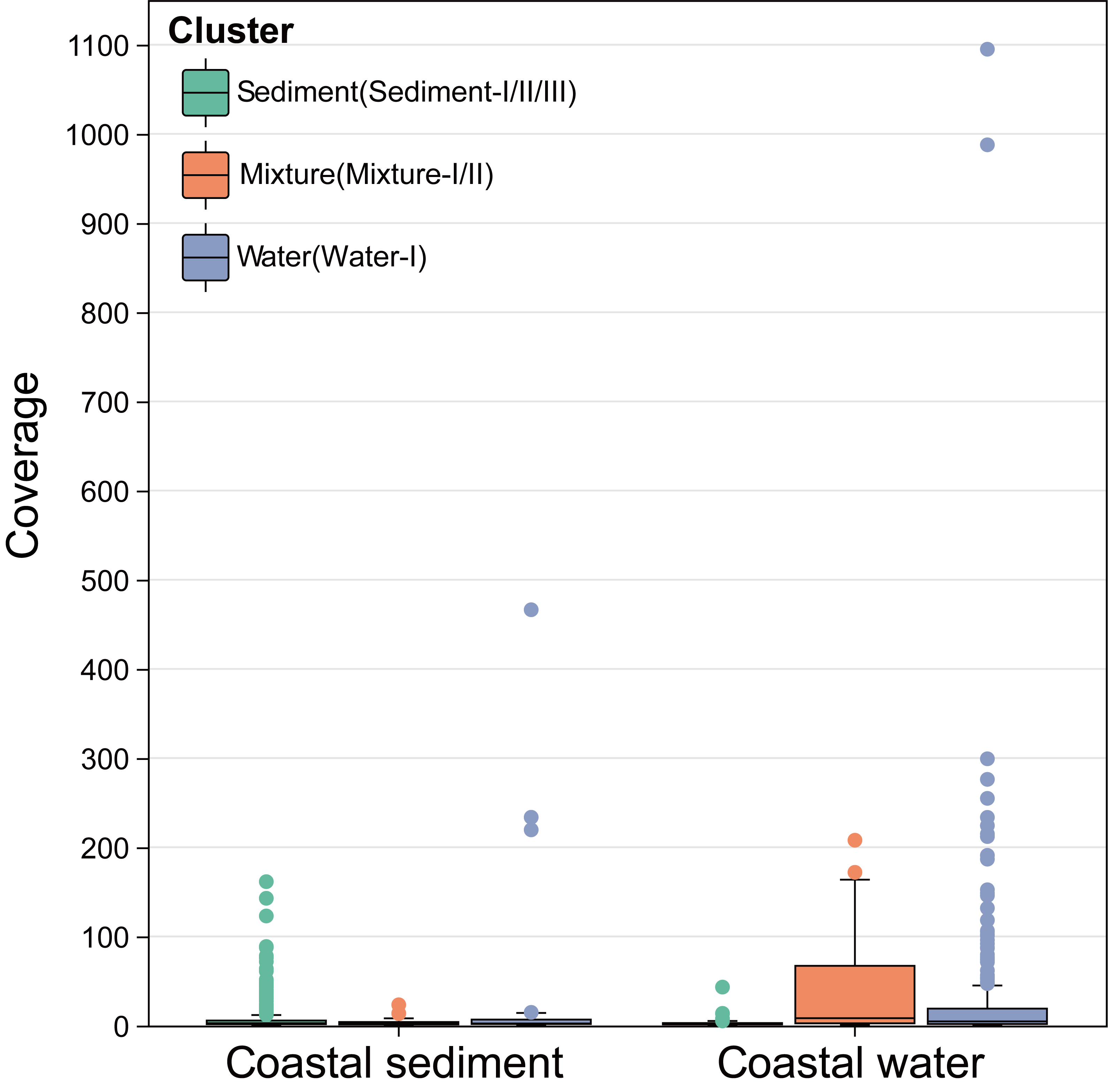
**

**Figure S24** Genome coverage of coastal *Nitrososphaeria* genomes across coastal sediment and coastal water samples. Genomes were grouped by source cluster: Sediment-derived (Sediment-I/II/III), Mixture (Mixture-I/II), and Water-derived (Water-I). Boxplots show average genome coverage for each genome within the two environments. In sediment samples, no significant differences were observed in coverage between genome origin groups (Kruskal–Wallis *P* = 0.288), while water samples showed highly source-specific coverage patterns (*P* < 1 × 10⁻¹¹).


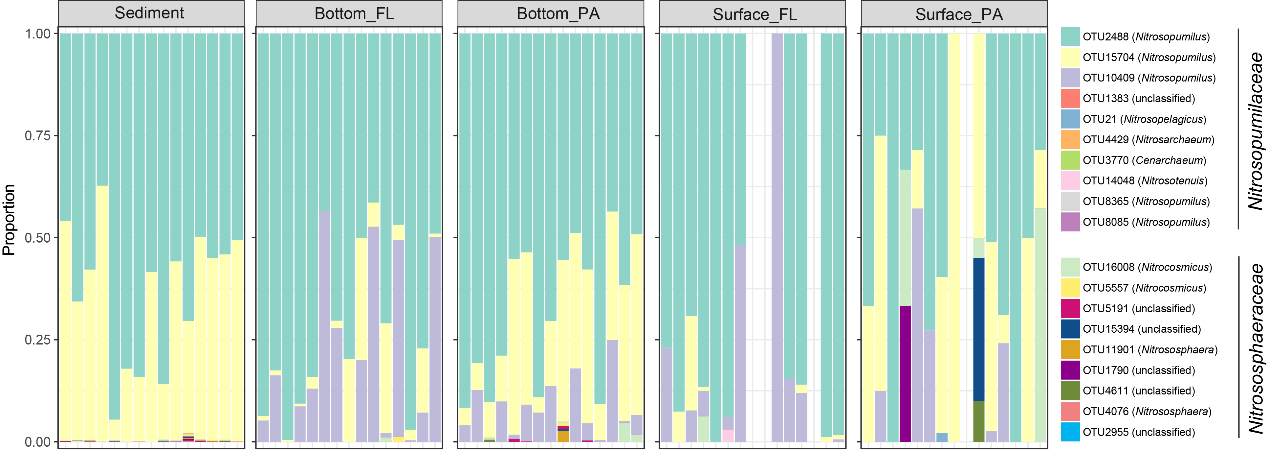


**Figure S25** The distribution of *Nitrososphaeria*-associated OTUs in the water and sediments of the Bohai Sea. FL, free-living; PA, particle-attached. Surface, surface water; Bottom, bottom water

**References**

1. Seitz KW, Dombrowski N, Eme L, Spang A, Lombard J, Sieber JR, *et al*. Asgard archaea capable of anaerobic hydrocarbon cycling. *Nat Commun*. 2019;**10**:1822.

2. Huerta-Cepas J, Forslund K, Coelho LP, Szklarczyk D, Jensen LJ, von Mering C, *et al.* Fast genome-wide functional annotation through orthology assignment by eggNOG-mapper. *Mol Biol Evol*. 2017;**34**:2115-22.

3. Capella-Gutiérrez S, Silla-Martínez JM, Gabaldón T. trimal: A tool for automated alignment trimming in large-scale phylogenetic analyses. *Bioinformatics*. 2009;**25**:1972-3.

4. Nguyen LT, Schmidt HA, von Haeseler A, Minh BQ. IQ-TREE: A fast and effective stochastic algorithm for estimating maximum-likelihood phylogenies. *Mol Biol Evol*. 2015;**32**:268-74.

5. Kalyaanamoorthy S, Minh BQ, Wong TK, von Haeseler A, Jermiin LS*.* ModelFinder: Fast model selection for accurate phylogenetic estimates. *Nat Methods*. 2017;**14**:587-9.

6. Ronquist F, Huelsenbeck JP. MrBayes 3: Bayesian phylogenetic inference under mixed models. *Bioinformatics*. 2003;**19**:1572-74.

7. Bengtsson-Palme J, Hartmann M, Eriksson KM, Pal C, Thorell K, Larsson DGJ, *et al.* Metaxa2: Improved identification and taxonomic classification of small and large subunit rrna in metagenomic data. *Mol Ecol Resour*. 2015;**15**:1403-14.

8. Quast C, Pruesse E, Yilmaz P, Gerken J, Schweer T, Yarza P, *et al.* The SILVA ribosomal RNA gene database project: Improved data processing and web-based tools. *Nucleic Acids Res*. 2012;**41**:D590-6.

9. Bolyen E, Rideout JR, Dillon MR, Bokulich NA, Abnet CC, Al-Ghalith GA, *et al.* Reproducible, interactive, scalable and extensible microbiome data science using QIIME 2. *Nat Biotechnol*. 2019;**37**:852-7.

10. Katoh K, Standley DM. MAFFT multiple sequence alignment software version 7: Improvements in performance and usability. *Mol Biol Evol*. 2013;**30**:772-80.

11. Wang H, Bagnoud A, Ponce-Toledo RI, Kerou M, Weil M, Schleper C, *et al.* Linking 16S rRNA gene classification to *amoA* gene taxonomy reveals environmental distribution of ammonia-oxidizing archaeal clades in peatland soils. *mSystems*. 2021:e0054621.

12. Kanehisa M, Sato Y, Morishima K. BlastKOALA and GhostKOALA: Kegg tools for functional characterization of genome and metagenome sequences. *J Mol Biol*. 2016;**428**:726-31.

13. Zhang H, Yohe T, Huang L, Entwistle S, Wu P, Yang Z, *et al.* dbCAN2: A meta server for automated carbohydrate-active enzyme annotation. *Nucleic Acids Res*. 2018;**46**:W95-W101.

14. Saier MH Jr, Reddy VS, Moreno-Hagelsieb G, Hendargo KJ, Zhang Y, Iddamsetty V, *et al.* The transporter classification database (TCDB): 2021 update. *Nucleic Acids Res*. 2021;**49**:D461-D467.

15. Wang X, Liu J, Li B, Liang J, Sun H, Zhou S, *et al*. Spatial heterogeneity of Vibrio spp. in sediments of Chinese marginal seas. *Appl Environ Microbiol*. 2019;**85**:e03064-18.

16. Park SJ, Kim JG, Jung MY, Kim SJ, Cha IT, Ghai R, *et al.* Draft genome sequence of an ammonia-oxidizing archaeon, “*Candidatus* Nitrosopumilus sediminis” AR2, from Svalbard in the Arctic Circle. *J Bacteriol.* 2012;194.

17. Qin W, Heal KR, Ramdasi R, Kobelt JN, Martens-Habbena W, Bertagnolli AD, *et al.* *Nitrosopumilus maritimus* gen. nov., sp. nov., *Nitrosopumilus cobalaminigenes* sp. nov., *Nitrosopumilus oxyclinae* sp. nov., and *Nitrosopumilus ureiphilus* sp. nov., four marine ammonia-oxidizing archaea of the phylum Thaumarchaeota. *Int J Syst Evol Microbiol*. 2017;**67**:5067–79.

18. Chen YJ, Leung PM, Cook PL, Wong WW, Hutchinson T, Eate V, *et al.* Hydrodynamic disturbance controls microbial community assembly and biogeochemical processes in coastal sediments. *ISME J*. 2022;**16**:750-63.

19. Mosier AC, Allen EE, Kim M, Ferriera S, Francis CA. Genome sequence of “*Candidatus* Nitrosopumilus salaria” BD31, an ammonia-oxidizing archaeon from the San Francisco Bay estuary. *J Bacteriol*. 2012;194.

20. Hugerth LW, Larsson J, Alneberg J, Lindh MV, Legrand C, Pinhassi J, *et al.* Metagenome-assembled genomes uncover a global brackish microbiome. *Genome Biol*. 2015;**16**:1-18.

21. Cabello-Yeves PJ, Zemskaya TI, Rosselli R, Coutinho FH, Zakharenko AS, Blinov VV, *et al.* Genomes of novel microbial lineages assembled from the sub-ice waters of lake baikal. *Appl Environ Microbiol*. 2018;**84**:e02132-17.

22. Orellana LH, Francis TB, Krüger K, Teeling H, Müller MC, Fuchs BM, *et al.* Niche differentiation among annually recurrent coastal marine group II Euryarchaeota. *ISME J*. 2019;**13**:3024-36.

23. Mehrshad M, Amoozegar MA, Ghai R, Shahzadeh Fazeli SA, Rodriguez-Valera F. Genome reconstruction from metagenomic data sets reveals novel microbes in the brackish waters of the Caspian Sea. *Appl Environ Microbiol*. 2016;**82**:1599-612.

24. Haro-Moreno JM, López-Pérez M, de la Torre JR, Picazo A, Camacho A, Rodriguez-Valera F. Fine metagenomic profile of the mediterranean stratified and mixed water columns revealed by assembly and recruitment. *Microbiome*. 2018;**6**:1-19.

25. Zou D, Wan R, Han L, Xu MN, Liu Y, Liu H, *et al*. Genomic characteristics of a novel species of ammonia-oxidizing archaea from the Jiulong River estuary. *Appl Environ Microbiol*. 2020;**86**:e00736–20.

26. Ahlgren NA, Chen Y, Needham DM, Parada AE, Sachdeva R, Trinh V, *et al.* Genome and epigenome of a novel marine Thaumarchaeota strain suggest viral infection, phosphorothioation DNA modification and multiple restriction systems. *Environ Microbiol*. 2017;**19**:2434-52.

27. Tian RM, Zhang W, Cai L, Wong YH, Ding W, Qian PY*.* Genome reduction and microbe-host interactions drive adaptation of a sulfur-oxidizing bacterium associated with a cold seep sponge. *mSystems*. 2017;**2**:10-1128.

28. Zou D, Li Y, Kao SJ, Liu H, Li M. Genomic adaptation to eutrophication of ammonia-oxidizing archaea in the Pearl River Estuary. *Environ Microbiol*. 2019;**21**:2320–32.

29. Kitzinger K, Padilla CC, Marchant HK, Hach PF, Herbold CW, Kidane AT, *et al.* Cyanate and urea are substrates for nitrification by Thaumarchaeota in the marine environment. *Nat Microbiol*. 2019;**4**:234-43.

30. Nakagawa T, Koji M, Hosoyama A, Yamazoe A, Tsuchiya Y, Ueda S, *et al.* *Nitrosopumilus zosterae* sp. Nov., an autotrophic ammonia-oxidizing archaeon of phylum Thaumarchaeota isolated from coastal eelgrass sediments of japan. *Int J Syst Evol Microbiol.* 2021;**71**:004961.

31. Matsutani N, Nakagawa T, Nakamura K, Takahashi R, Yoshihara K, Tokuyama T. Enrichment of a novel marine ammonia-oxidizing archaeon obtained from sand of an eelgrass zone. *Microbes Environ*. 2011;**26**:23-9.

32. Qin W, Amin SA, Martens-Habbena W, Walker CB, Urakawa H, Devol AH, *et al.* Marine ammonia-oxidizing archaeal isolates display obligate mixotrophy and wide ecotypic variation. *Proc Natl Acad Sci U S A*. 2014;**111**:12504-9.

33. Qin W, Heal KR, Ramdasi R, Kobelt JN, Martens-Habbena W, Bertagnolli AD, *et al.* *Nitrosopumilus maritimus* gen. Nov., sp. Nov., *Nitrosopumilus cobalaminigenes* sp. Nov., *Nitrosopumilus oxyclinae* sp. Nov., and *Nitrosopumilus ureiphilus* sp. Nov., four marine ammonia-oxidizing archaea of the phylum Thaumarchaeota. *Int J Syst Evol Microbiol*. 2017;**67**:5067-79.

34. Bayer B, Vojvoda J, Offre P, Alves RJ, Elisabeth NH, Garcia JA, *et al.* Physiological and genomic characterization of two novel marine thaumarchaeal strains indicates niche differentiation. *ISME J*. 2016;**10**:1051-63.

35. Bayer B, Vojvoda J, Reinthaler T, Reyes C, Pinto M, Herndl GJ. *Nitrosopumilus adriaticus* sp. Nov. And *Nitrosopumilus piranensis* sp. Nov., two ammonia-oxidizing archaea from the adriatic sea and members of the class *Nitrososphaeria*. *Int J Syst Evol Microbiol*. 2019;**69**:1892-902.

36. Kim JG, Park SJ, Sinninghe Damsté JS, Schouten S, Rijpstra WIC, Jung MY, *et al.* Hydrogen peroxide detoxification is a key mechanism for growth of ammonia-oxidizing archaea. *Proc Natl Acad Sci U S A*. 2016;**113**:7888-93.

37. Könneke M, Bernhard AE, de la Torre JR, Walker CB, Waterbury JB, Stahl DA. Isolation of an autotrophic ammonia-oxidizing marine archaeon. *Nature*. 2005;**437**:543-46.

38. Zhong H, Lehtovirta-Morley L, Liu J, Zheng Y, Lin H, Song D, *et al.* Novel insights into the Thaumarchaeota in the deepest oceans: Their metabolism and potential adaptation mechanisms. *Microbiome*. 2020;**8**:78.

39. Wang Y, Huang JM, Cui GJ, Nunoura T, Takaki Y, Li WL, *et al.* Genomics insights into ecotype formation of ammonia-oxidizing archaea in the deep ocean. *Environ Microbiol*. 2019;**21**:716-29.

40. Probst AJ, Ladd B, Jarett JK, Geller-McGrath DE, Sieber CM, Emerson JB, *et al.* Differential depth distribution of microbial function and putative symbionts through sediment-hosted aquifers in the deep terrestrial subsurface. *Nat Microbiol*. 2018;**3**:328-36.

41. Jung MY, Islam MA, Gwak JH, Kim JG, Rhee SK. *Nitrosarchaeum koreense* gen. nov., sp. nov., an aerobic and mesophilic, ammonia-oxidizing archaeon member of the phylum *Thaumarchaeota* isolated from agricultural soil. *Int J Syst Evol Microbiol*. 2018;**68**:3084-95.

42. Jung MY, Park SJ, Min D, Kim JS, Rijpstra WIC, Sinninghe Damsté JS, *et al.* Enrichment and characterization of an autotrophic ammonia-oxidizing archaeon of mesophilic crenarchaeal group i. 1a from an agricultural soil. *Appl Environ Microbiol*. 2011;**77**:8635-47.

43. Blainey PC, Mosier AC, Potanina A, Francis CA, Quake SR. Genome of a low-salinity ammonia-oxidizing archaeon determined by single-cell and metagenomic analysis. *PloS* *One*. 2011;**6**:e16626.

44. Mosier AC, Allen EE, Kim M, Ferriera S, Francis CA. Genome sequence of “*Candidatus* Nitrosoarchaeum limnia” BG20, a low-salinity ammonia-oxidizing archaeon from the san francisco bay estuary. *J Bacteriol*. 2012;194.

45. Hallam SJ, Konstantinidis KT, Putnam N, Schleper C, Watanabe YI, Sugahara J, *et al.* Genomic analysis of the uncultivated marine crenarchaeote *Cenarchaeum symbiosum*. *Proc Natl Acad Sci U S A.* 2006;**103**:18296-301.

46. Hallam SJ, Mincer TJ, Schleper C, Preston CM, Roberts K, Richardson PM, *et al.* Pathways of carbon assimilation and ammonia oxidation suggested by environmental genomic analyses of marine *Crenarchaeota*. *PLoS Biol*. 2006;**4**:e95.

47. Hug LA, Thomas BC, Sharon I, Brown CT, Sharma R, Hettich RL, *et al.* Critical biogeochemical functions in the subsurface are associated with bacteria from new phyla and little studied lineages. *Environ Microbiol*. 2016;**18**:159-73.

48. Kerou M, Ponce-Toledo RI, Zhao R, Abby SS, Hirai M, Nomaki H, *et al.* Genomes of Thaumarchaeota from deep sea sediments reveal specific adaptations of three independently evolved lineages. *ISME J*. 2021;**15**:2792-808.

49. Rinke C, Schwientek P, Sczyrba A, Ivanova NN, Anderson IJ, Cheng JF, *et al.* Insights into the phylogeny and coding potential of microbial dark matter. *Nature*. 2013;**499**:431-7.

50. Lehtovirta-Morley LE, Stoecker K, Vilcinskas A, Prosser JI, Nicol GW. Cultivation of an obligate acidophilic ammonia oxidizer from a nitrifying acid soil. *Proc Natl Acad Sci U S A*. 2011;**108**:15892-7.

51. Lebedeva EV, Hatzenpichler R, Pelletier E, Schuster N, Hauzmayer S, Bulaev A, *et al.* Enrichment and genome sequence of the group i. 1a ammonia-oxidizing archaeon “*Ca.* Nitrosotenuis uzonensis” representing a clade globally distributed in thermal habitats. *PLoS One*. 2013;**8**:e80835.

52. Li Y, Ding K, Wen X, Zhang B, Shen B, Yang Y. A novel ammonia-oxidizing archaeon from wastewater treatment plant: Its enrichment, physiological and genomic characteristics. *Sci Rep*. 2016;**6**:23747.

53. Lehtovirta-Morley LE, Sayavedra-Soto LA, Gallois N, Schouten S, Stein LY, Prosser JI, *et al.* Identifying potential mechanisms enabling acidophily in the ammonia-oxidizing archaeon “*Candidatus* Nitrosotalea devanaterra”. *Appl Environ Microbiol*. 2016;**82**:2608-19.

54. Tourna M, Stieglmeier M, Spang A, Könneke M, Schintlmeister A, Urich T, *et al.* *Nitrososphaera viennensis*, an ammonia oxidizing archaeon from soil. *Proc Natl Acad Sci U S A*. 2011;**108**:8420-5.

55. Stieglmeier M, Klingl A, Alves RJ, Rittmann SKM, Melcher M, Leisch N, *et al.* *Nitrososphaera viennensis* gen. nov., sp. nov., an aerobic and mesophilic, ammonia-oxidizing archaeon from soil and a member of the archaeal phylum *Thaumarchaeota*. *Int J Syst Evol Microbiol*. 2014;**64**:2738-52.

56. Zhalnina KV, Dias R, Leonard MT, Dorr de Quadros P, Camargo FA, Drew JC, *et al.* Genome sequence of *Candidatus* Nitrososphaera evergladensis from group I.1b enriched from everglades soil reveals novel genomic features of the ammonia-oxidizing archaea. *PLoS One*. 2014;**9**:e101648.
